# Melatonin Ameliorate neuroinflammation in activated microglia through the Aryl hydrocarbon-Nrf2 axis

**DOI:** 10.7150/ijbs.105081

**Published:** 2025-06-09

**Authors:** Meei-Ling Sheu†, Cheng-Ning Yang, Liang-Yi Pan, Jason Sheehan, Liang-Yu Pan, Weir-Chiang You, Chien-Chia Wang, Ying Ju Chen, Hong-Shiu Chen†, Hung-Chuan Pan

**Affiliations:** 1 Institute of Biomedical Science, National Chung-Hsing University, Taichung, Taiwan.; 2 Doctoral Program in Biotechnology Industrial Management and Innovation, National Chung Hsing University, Taichung, Taiwan.; 3 Rong Hsing Research Center for Translational Medicine, National Chung Hsing University, Taichung, Taiwan.; 4 Department of Medical Research, Taichung Veterans General Hospital, Taichung, Taiwan.; 5 Ph.D. Program in Translational Medicine, Rong Hsing Research Center for Translational Medicine, National Chung Hsing University, Taichung, Taiwan.; 6 Department of Dentistry, School of Dentistry, College of Medicine, National Taiwan University, Taipei, Taiwan.; 7 Faculty of Medicine, Kaohsiung Medical University, Kaohsiung, Taiwan.; 8 Department of Neurosurgery, University of Virginia, Charlottesville, VA, USA.; 9 Faculty of Medicine, Poznan University of Medical Sciences, Poland.; 10 Department of Radiation Oncology, Taichung Veterans General Hospital, Taichung.; 11 Department of Life Sciences, National Central University, Taoyuan, Taiwan.; 12 PhD program in Health and Social Welfare for Indigenous Peoples, Providence University, Taichung, Taiwan.; 13 Department of Neurosurgery, Tungs' Taichung Metro-Harbor Hospital, Taichung 40210, Taiwan.; 14 Department of Neurosurgery, Neurological Institute, Taichung Veterans General Hospital, Taichung, Taiwan.

**Keywords:** Melatonin, Microglia, Aryl hydrocarbon receptors, Nrf2

## Abstract

Microglia-mediated neuroinflammation is central to many neurological disorders. The Aryl hydrocarbon receptor (AhR) is highly expressed in microglia and plays a key role in neuroinflammation. While melatonin has anti-inflammatory effects in neurodegenerative disorders, its connection to AhR in modulating neuroinflammation is unclear. This study found that melatonin inhibits NF-κB activity, reduces pro-inflammatory mediators, and promotes an M2 microglia profile. Melatonin also enhances phospho-AhR (Tyr239) activation, increases Nrf2 expression, and decreases LPS-induced ROS generation in microglia. Melatonin's effects are similar to those achieved by AhR activation. In contrast, AhR knockout worsens neurological deficits and microglial activation, while melatonin reverses these effects by restoring AhR expression. In conclusion, effects of melatonin on microglia-mediated neuroinflammation are closely linked to phospho-AhR (Tyr239) activation and its associated downstream gene, Nrf2, via the AhR/Nrf2/ARE pathway. Therefore, melatonin, in conjunction with AhR may offer promising therapeutic benefits in neuroinflammatory disorders.

## Introduction

Microglial activation is a key component of neuroinflammation, acting as the central nervous system's immune defense against pathogens and injury 1. Under normal conditions, microglia have a ramified morphology for efficient surveillance, synapse pruning, and clearance of apoptotic neurons to maintain homeostasis 2. When homeostasis is disrupted, microglia change phenotypes to support neurogenesis and tissue remodeling in response to trauma, inflammation, and stroke 3-7.

The Aryl hydrocarbon receptor (AhR), a PAS family member, includes a basic helix-loop-helix domain and associates with HSP90, XAP2, p23, and pp60src 8. AhR regulates neural functions and responds to xenobiotics like TCDD, affecting neuronal proliferation, differentiation, and survival 9. AhR is widely expressed in the brain, including in microglia, astrocytes, and endothelial cells, and it is implicated in neurodegenerative disorders 10, 11. This receptor's expression supports various neurodegenerative disorders through both direct and indirect effects, whereas its downregulation conveys a detrimental response 7, 12-15. When activated, AhR moves to the nucleus to bind genes with antioxidant response elements, promoting detoxification and antioxidant genes like Nrf2, HO-1, NQO1, and GST 16, 17. AhR signaling helps defend against oxidative stress and suppress inflammation. AhR deficiency in mice enhances NF-κB activity and increases inflammasome activity of NLRP3 18,19. AhR also modulates inflammatory responses by influencing p65 activation and interacting with Nrf2 signaling 20, 21.

Melatonin, primarily secreted by the pineal gland, acts through M1 and M2 receptors as an antioxidant, anti-inflammatory, antidepressant, and antitumor agent 22-25. It reduces pro-inflammatory cytokines (TNFα, IL-6, IL1β) in response to LPS, mitigates mitochondrial toxicity, prevents apoptosis, and promotes autophagy 26,27. Melatonin shifts LPS-induced microglial polarization from the pro-inflammatory M1 to the anti-inflammatory M2 phenotype 28. Previous research showed melatonin stimulates the ER stress pathway, inhibits epithelial-mesenchymal transition 29, and protects the hippocampal dentate gyrus from LPS-induced damage via the SIRT1/Nrf2 pathway 29-31.

Given AhR's distribution and regulation alongside the Nrf2 pathway as well as its similar response to oxidative stress as melatonin, a potential link between AhR and melatonin is suggested. This study uses docking systems and in-vitro and *in*-*vivo* experiments to explore the interaction between AhR and melatonin in activated microglial cells.

## Material and methods

### Cell culture

Cell Culture C57BL/6 mice (4-5 weeks old, 20-22 g) were obtained from National Applied Research Laboratories (Taipei, Taiwan), and AhR-knockout mice (6-8 weeks old, 18-22 g) were secured from Jackson Laboratory (Bar Harbor, ME, USA). Microglial cells were cultured from the cerebral cortices of these mice, following established protocols 6, 7, 30. Primary microglia cells were cultured from newborn mouse pups of the cerebral cortices within 3 days-old mice. To collect microglia at the 7-day time point, we mechanically dissociated cells by vigorously tap the flasks on the bench top and collect the floating cells in conditioned culture media. The resulting cells are purified microglia. These cells were seeded and plated onto coverslips in 24-well plates at a density of 1.5 x 10^5^ cells/coverslip, and they were cultured in Eagle's minimal essential medium (MEM) supplemented with 5% horse serum, 5% fetal bovine serum (FBS), 21 mM glucose, 26.5 mM bicarbonate, and 2 mM L-glutamine. Purity of primary microglial cells (>97%) was confirmed by Iba1 staining. BV-2 immortalized microglial cell line was cultured in DMEM with 10% heat-inactivated low-endotoxin FBS and antibiotics in a 5% CO2 atmosphere. In addition, a BV-2 immortalized microglial cell line from Yen Jen Sung (Institute of Anatomy and Cell Biology, School of Life Science, National Yang-Ming University, Taipei, Taiwan) was also utilized.

### Stereotaxic surgery and drug injection

C57BL/6J mice (25-30 g), either wild-type or AhR (-/-), were anesthetized with chloral hydrate (400 mg/kg i.p.) and secured in a stereotaxic frame. A stereotactic injection of LPS (10 μg) was administered into the right cerebral cortex of either wild or AhR-deleted mice, combined with melatonin (5 mg/kg) in a total of 20 μL PBS 6, 7, 32. The melatonin dosage was based on our previous investigation 28. The injection was delivered at a rate of 1 μL/min using a 26-gauge Hamilton syringe needle, which remained in place for 5 minutes post-injection to prevent reflux.

### Immunohistochemistry

Primary microglia or BV-2 cells were treated and fixed for immunohistochemical staining. Animals were perfused with saline containing 0.5% sodium nitrate and heparin (10 U/mL), followed by 4% paraformaldehyde in 0.1 M phosphate buffer. Tissues were post-fixed, washed in PBS, cryoprotected in 30% sucrose, and sectioned at 40 μm thickness 32. Sections were incubated with primary antibodies (Supplementary Table I) overnight at room temperature, followed by incubation with FITC- or Texas Red-labeled secondary antibodies (Supplementary Table II), then mounted for imaging using an Olympus IX71 confocal laser scanning microscope (CLSM).

### Western blot analysis

Protein expressions were assessed using Western blotting, following established protocols 33. Briefly, proteins (60 μg) were separated by SDS-PAGE, transferred to nitrocellulose membranes, and blocked for 1 hour in phosphate-buffered saline with Tween 20 (0.1%) and non-fat milk (5%). Membranes were then incubated with primary antibodies (Supplementary Table I) for 1 hour. Following washing, membranes were incubated with horseradish peroxidase-conjugated secondary antibodies (Supplementary Table II) for 1 hour, and chemiluminescence signals were detected using commercial reagents (Amersham Biosciences, Amersham, UK).

### Griess assay

The concentration of nitrite in cell culture supernatants was quantified using the nitrite/nitrate colorimetric assay kit (R & D Systems, Minneapolis, MN, USA). Nitrite, a byproduct of NO oxidation, served as an indicator of NO production. The Griess reagent was employed to measure nitrite levels, and absorbance was read at 550 nm using a thermomicroplate reader (Molecular Devices, San Jose, CA, USA) 33.

### STRING

Open website STRING dataset is a proteomic data base focusing on the networks and interactions of proteins in a wide array of different species. We employed the STRING database to analyze protein-protein interaction networks and perform functional enrichment analyses for a selected genome.

### LIGPLOT

LigPlot, utilizing the LIGPLOT program, automatically generates 2D diagrams illustrating interactions between ligands and proteins. These diagrams depict hydrogen bonds and hydrophobic contacts involving the protein's main-chain or side-chain elements. LigPlot can visualize interactions for sets of ligands binding to the same protein target, a single ligand binding to homologous proteins, or cases where both the protein and ligand vary. Additionally, it provides links to view 3D representations of these diagrams in PyMOL or RasMol, highlighting all interactions.

### Molecular docking modeling

PyMOL is a widely used macromolecular visualization software that utilizes the OpenGL Extension Wrangler Library (GLEW) and Free OpenGL Utility Toolkit (Freeglut). It employs a cross-platform widget toolkit (Tk) for GUI elements and can generate high-quality images and movies depicting macromolecules in diverse representations like ribbon, cartoon, dot, surface, sphere, stick, and line. PyMOL extends its capabilities to include protein-ligand modeling, molecular simulations (MS), and virtual screening (VS) units. Its computational drug discovery tools have been effectively employed to identify potential new drug candidates across various targets 34.

### ELISA

The levels of IL-1β, IL-6, TNF-α, IL-4, IL-10, TGF-β, NF-κB binding activity (assessed using the TransAM® NFκB kit), and Nrf2 DNA-binding activity were measured using commercially available kits listed in Supplementary Table III. ELISA assays were performed with equal amounts of protein following the manufacturer's instructions.

### EthoVision XT with novel object test

The novel object recognition test was utilized to evaluate visual memory in rodents following established protocols 35. Animals underwent three phases: In the first phase, they were allowed to habituate to an empty arena for 10 minutes. In the second phase, after a 15-minute period in their home cage, animals were placed in the arena equidistant from two identical objects and observed for 3 minutes. In the final phase, one object was replaced with a novel object, and animals were reintroduced to the arena for another 3-minute observation period. The test assessed short-term and long-term memory at 1 hour and 24 hours post exposure to the novel objects, respectively.

### Open field locomotion test

The open field test involves placing an animal in a large cubic box (usually 1 m in length, width, and height) with an open top. The animal is positioned in the center of the bottom surface, and its activities are recorded for minutes to hours as it explores the environment. Afterward, computerized tracking systems analyze the animal's movements, assessing horizontal activity, time spent in different zones within the field, and total distance covered during the observation period.

### RNA isolation and quantitative real-time PCR

To analyze gene expression, RNA was extracted from BV2 cell lines, primary microglia cultures, or brain cortical tissue using Trizol reagent. Purified RNA was then reverse-transcribed into cDNA using a standard cDNA synthesis kit. Quantitative real-time polymerase chain reaction (qPCR) was performed using SYBR Advantage qPCR Premix (Clontech, Mountain View, California) and oligonucleotide primers listed in Supplementary Table IV. Oligonucleotide synthesis was conducted by MWG-Biotech AG (Ebersberg, Germany). For IL-1β, iNOS, CD206, and IL-10 gene expression, PCR conditions included an initial denaturation at 95°C for 3 min, followed by 40 cycles of 95°C for 30 s, 60°C for 1 min 30 s, and a melting curve analysis with a final step of 0.5°C temperature change. Reactions were performed in triplicate using an Applied Biosystems StepOne Real-time PCR system.

### Arginase activity

Arginase activity in cell lysates was assessed according to established protocols 36. After treatments, cells were washed twice with ice-cold Dulbecco's PBS and then lysed in 300 μl of lysis buffer using sonication (20 kHz, 30 s with 10 s/cycle). Subsequently, the lysates were heated at 55-60°C for 10 minutes to activate arginase. The hydrolysis of L-arginine by arginase was initiated by incubating the activated lysates with 50 μl of L-arginine (0.5 M, pH 9.7) at 37°C for 1 hour, followed by quenching with 400 μl of an acid solution (H2SO4:H3PO4, 1:3:7). Urea production, indicative of arginase activity, was determined colorimetrically by adding 25 μl of α-isonitrosopropiophenone (9% in absolute ethanol) and heating the mixture at 100°C for 45 minutes. After 10 minutes of dark incubation at room temperature, urea concentration was quantified at 550 nm using a microplate reader (Molecular Devices, Sunnyvale, CA).

### ROS production

ROS generation was assessed using 2,7-dichlorofluorescein diacetate (DCF-DA; Sigma-Aldrich, USA). Cells were seeded in 48-well plates (2x10^4^cells per well) and treated as indicated. Subsequently, cells were incubated with 10 μM DCF-DA and monitored over a specified time course. Qualitative assessment of intracellular ROS levels was performed using a fluorescence microscope (Olympus CX-41), while quantitative measurements were conducted using a multimode microplate fluorometer (excitation/emission 490/525 nm; PerkinElmer). In addition, the adherent cells were washed with a buffer and subsequently incubated with DCFDA for 45 minutes. After another wash with the buffer, fluorescent microscopy was used for analysis under low-light conditions to minimize photo-bleaching.

### Electrophoretic mobility shift assay (EMSA)

The electrophoretic mobility shift assay (EMSA) was conducted following established protocols 9. The electrophoretic mobility shift assay (EMSA) was also performed following established protocols. Oligonucleotide probes with the AhR consensus binding sequence (-650 to -625, 5′-GCACGAGTTTGCAGCGTGGACTC-3′) were labeled according to manufacturer instructions. Each binding reaction included 2 ng of labeled oligonucleotide, 2 μg of poly dIdC (Amersham Pharmacia Biotech) carrier, and 2 μg of nuclear protein in a binding buffer (10 mM HEPES, 60 mM KCl, 1 mM DTT, 1 mM EDTA, 7% glycerol, pH 7.6) incubated for 30 minutes at room temperature. DNA-protein complexes were separated on 6% non-denaturing polyacrylamide gels and visualized using autoradiographic films.

### Luciferase reporter assay

Cells at 60% confluence were co-transfected with 0.2 μg of the NFκB or ARE promoter-reporter construct and 0.05 μg of the pRLTK vector, which drives Renilla luciferase expression under the control of a thymidine kinase promoter (Promega, Mannheim, Germany). The pRL-tk-LUC vector serves to normalize for transfection efficiency. Following transfection, cells were lysed and processed using the Dual-Luciferase Assay Kit (Promega) according to the manufacturer's protocol. Luciferase activity was measured using a luminometer (LKB, Rockville, MD, USA) and normalized against Renilla luciferase activity to account for variations in transfection efficiency.

### Transfection of AhR in BV-2 cells

Primary microglia and BV2 microglial cell lines were seeded into 6-well plates at a density of 2x10^5^ cells per well and maintained in macrophage medium. After 72 hours of culture, viral particles were added at multiplicities of infection (MOI) ranging from 1 to 100, along with polybrene (8 μg/ml). Cells were then cultured for an additional 72 hours, during which enhanced green fluorescent protein (eGFP) expression was assessed using fluorescence microscopy. Subsequently, cells were washed with PBS and switched to serum-free medium for the indicated treatments.

### Ligand binding assay

This assay was performed as previously described, using a 10 mM 2-(N-morpholino) ethanesulfonic acid (MES) buffer containing 10 mM MnCl₂ and 1 mM EDTA, adjusted to pH 6.0 with KOH. Initial tissue linearity studies were conducted using 0.1-200 μg of cells containing recombinant human AhR (Abbexa Ltd, Catalogue No: abx065464) in a total volume of 0.5 ml within 96-well deep-well assay blocks (Matrix Technologies Corp., Hudson, NH, USA). [³H]-melatonin (PerkinElmer Inc.) was used as the radioligand. Proteins were rapidly thawed, diluted to the desired concentration in binding buffer, and mixed into a homogeneous suspension before dispensation. Following the addition of the radioligand, assay mixtures were incubated for 120 minutes on a rotary shaker (50 rpm). The reaction was terminated by rapid vacuum filtration using Whatman GF/B glass filter mats (pre-soaked in 0.5% polyethyleneimine) with cold MES buffer. Bound radioligand was quantified by liquid scintillation counting (Packard BioScience).

### Competition binding assays

To assess the specificity of [^3^H]-melatonin binding to the aryl hydrocarbon receptor (AhR), competition binding assays were performed using 2,3,7,8-tetrachlorodibenzo-p-dioxin (TCDD), a high-affinity AhR ligand. [^3^H]-melatonin was used as a radiolabeled tracer. AhR-containing cytosolic extracts were prepared from BV2. Binding reactions were carried out in a total volume of 100 µL in assay buffer containing buffer composition (10 mM HEPES, pH 7.9, 1.5 mM MgCl₂, 0.5 mM DTT, and 10% glycerol). The reactions were incubated at room temperature for 1 hour to allow equilibrium binding. [^3^H]-melatonin was added at a final concentration, and increasing concentrations of unlabeled TCDD ranging from 0.1 to 100 nM were included in parallel reactions to compete for binding to AhR. Control reactions without competitor were included to determine total binding. Following incubation, bound and free ligand were separated by charcoal adsorption, filtration through glass fiber filters, or gel filtration. Radioactivity associated with the receptor-bound fraction was measured using liquid scintillation counting. Each condition was run in triplicate, and experiments were repeated at least three times. Data were analyzed using nonlinear regression to determine the IC₅₀ value of TCDD, defined as the concentration of competitor that reduces specific [^3^H]-melatonin binding by 50%.

### Saturation binding assays

Saturation binding assays were conducted to determine the binding affinity (Kd) and the maximum binding capacity (Bmax) of [^3^H]-melatonin for the aryl hydrocarbon receptor (AhR). AhR-containing cytosolic extracts were prepared from BV2 as previously described. Binding reactions were set up in a final volume of 100 µL containing assay buffer (10 mM HEPES, pH 7.9, 1.5 mM MgCl₂, 0.5 mM DTT, 10% glycerol) and various concentrations of [^3^H]-melatonin as indicated to assess saturation. Samples were incubated at room temperature for 1 hour to reach equilibrium. Non-specific binding was determined in parallel samples containing a 100-fold excess of unlabeled melatonin. Bound and free radioligand were separated using filtration through glass fiber filters, or dextran-coated charcoal adsorption, and radioactivity was measured via liquid scintillation counting. Specific binding was calculated by subtracting non-specific from total binding. Data were analyzed by nonlinear regression using a one-site binding model to derive Kd and Bmax values.

### Gene silencing and mutagenesis

For gene silencing, wild-type cells were transfected with siRNA targeting AhR (shAhR) or a non-targeting control shRNA. The pLKO.1 empty vector was used as a negative control, in line with available TRC shRNA control standards. To investigate the role of Tyr239 in melatonin binding, a mutant AhR construct was generated by gene synthesis, in which tyrosine at position 239 was substituted with phenylalanine (Y239F). The Y239F mutant plasmid was synthesized by OMICS BIOTECHNOLOGY CO., LTD.

### Statistical analyses

The data in this study are expressed as mean ± standard error of the mean (SEM). Statistical analyses were conducted using ANOVA followed by Fisher's least significant difference test. Statistical significance was set at p-value < 0.05.

## Results

### Melatonin suppresses production of pro-inflammatory mediators and promotes expression of anti-inflammatory M2 markers in LPS-induced BV2 and primary microglia cells

**Figure 1:**
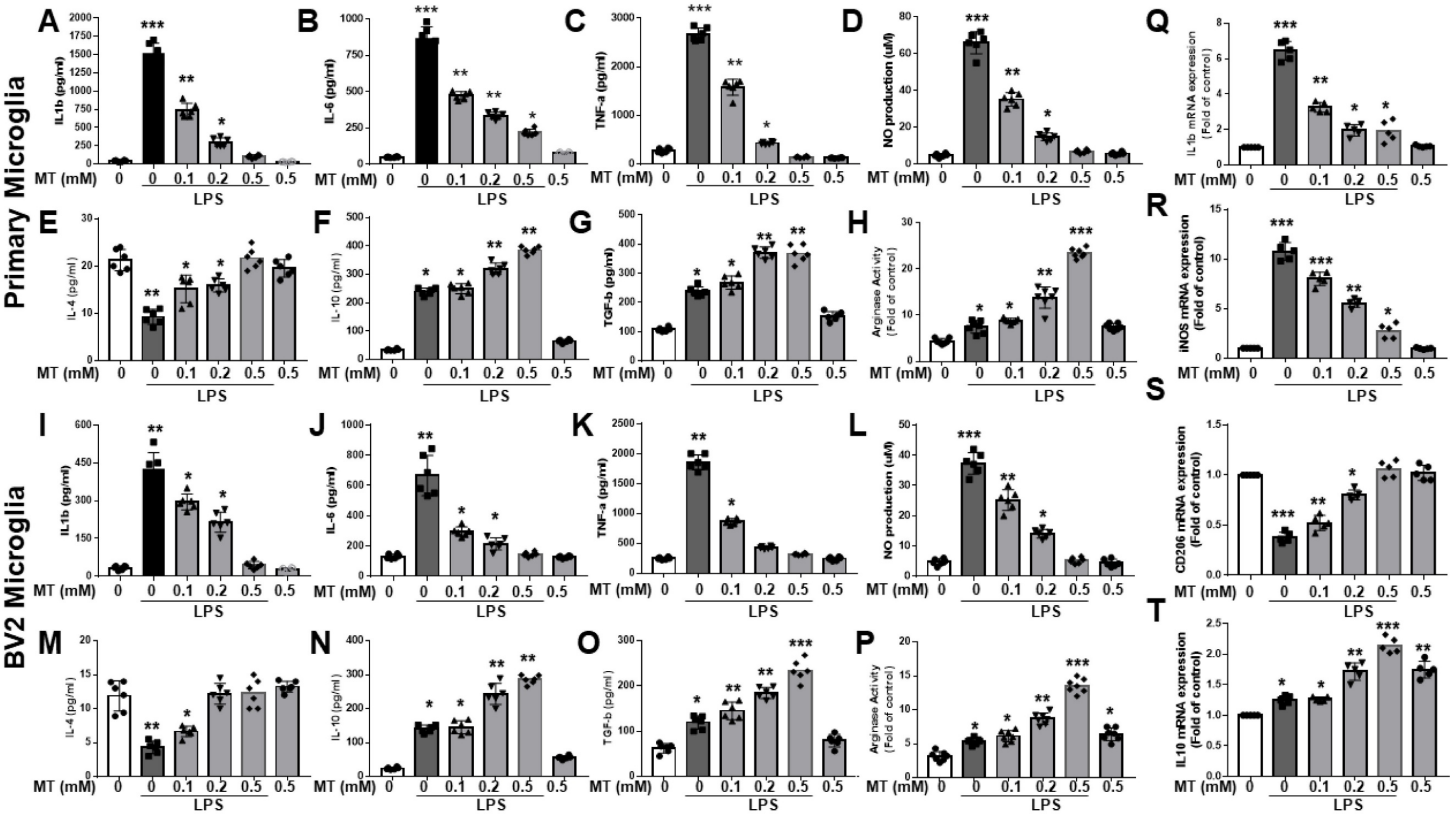
Attenuation of the inflammatory reaction and acceleration of anti-inflammatory response in LPS-activated microglia by melatonin. The LPS-treated BV2 or primary microglia cells are subjected to escalated dosage of melatonin treatment. The supernatants were obtained to determine inflammatory cytokine (IL-1β, IL-6, TNF-α) and anti-inflammatory cytokines (IL-4, IL-10, TGF-β, arginase activity) by ELISA. NO production was assessed by Griess assay. The harvested microglia cells were subjected to qRT-PCR to investigate the mRNA expression in IL-1β, iNOS, CD 206 and IL-10. (A) Determination of IL-1β expression in LPS treated BV2 in relation to escalating dosage of melatonin. (B) Determination of IL-6 expression in LPS treated BV2 cells in relation to escalating dosage of melatonin. (C) Determination of TNF-α expression in LPS treated BV2 cells in relation to escalating dosage of melatonin. (D) Measurement NO production in LPS-treated BV2 in relation to escalating dosage of melatonin. (E) Determination of IL-4 expression in LPS treated BV2 cells in relation to escalating dosage of melatonin. (F) Determination of IL-10 expression in LPS treated BV2 cells in relation to escalating dosage of melatonin. (G) Determination of TGF-β expression in LPS treated BV2 cells in relation to escalating dosage of melatonin. (H) Measurement of arginase activity in LPS treated BV2 cells in relation to escalating dosage of melatonin. (I) Determination of IL-1β expression in LPS treated primary microglia cells in relation to escalating dosage of melatonin. (J) Determination of IL-6 expression in LPS treated primary microglia cells in relation to escalating dosage of melatonin. (K) Determination of TNFα- expression in LPS treated primary microglia cells in relation to escalating dosage of melatonin. (L) Measurement NO production in LPS-treated primary microglia cells in relation to escalating dosage of melatonin. (M) Determination of IL-4 expression in LPS treated primary microglia cells in relation to escalating dosage of melatonin. (N) Determination of IL-10 expression in LPS treated primary microglia cells in relation to escalating dosage of melatonin. (O) Determination of TGF-β expression in LPS treated primary cultured microglia cells in relation to escalating dosage of melatonin. (P) Measurement of arginase activity in LPS treated primary microglia cells in relation to escalating dosage of melatonin. (Q) Expression of Il-1β mRNA in LPS treated BV2 cells in relation to escalating dosage of melatonin measured as a fold of increase related to control. (R) Expression of iNOS mRNA in LPS treated BV2 cells in relation to escalating dosage of melatonin measured as a fold of increase related to control. (S) Expression of CD 206 mRNA in LPS treated primary microglia cells in relation to escalating dosage of melatonin measured as a fold of increase related to control. (T) Expression of IL-10 mRNA in LPS treated primary cultured microglia cells in relation to escalating dosage of melatonin measured as a fold of increase related to control. N=6 in each independent experiment. Data analysis was done by performing one-way analysis of variance test followed by post hoc Tukey's test. Statistical significance: *p < 0.05; **p < 0.01; ***p<0.001, compared to control group.

Melatonin inhibited the production of LPS-stimulated pro-inflammatory cytokines (IL-1β, IL-6, and TNF-α) and nitric oxide (NO) in primary microglia and BV2 microglia cells in a dose-dependent manner (Fig. 1A-D, I-L). It also reduced mRNA expressions of IL-1β and iNOS in LPS-activated primary microglia cells and increased anti-inflammatory cytokines (IL-4, IL-10, TGF-β) and arginase activity (Fig. 1Q-R, E-H, M-P). Additionally, melatonin significantly elevated mRNA levels of M2 markers (CD206, IL-10) in a dose-dependent manner (Fig. 1S, T). Overall, melatonin inhibited pro-inflammatory M1 mediators and promoted anti-inflammatory M2 markers in LPS-stimulated microglia cells.

### Melatonin suppressed iNOS and COX-2 expressions, inactivated NF-κB (p65) as well as inhibited NF-κB (p65) translocation in LPS-induced BV2 and primary microglia cells

**Figure 2:**
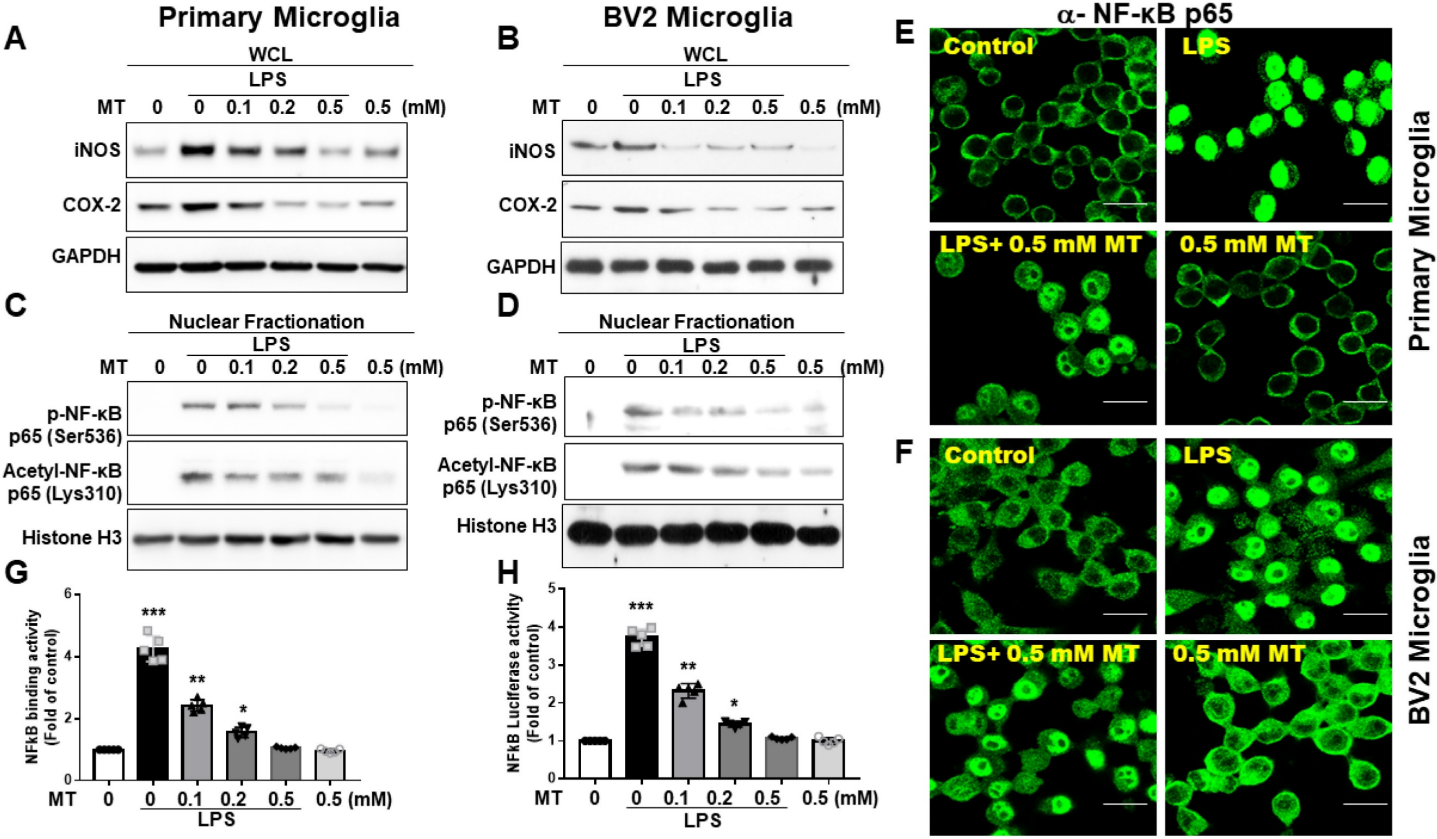
Regulation of NF-κB activity and downstream gene expression in LPS activated microglia cells. The LPS-treated BV-2 cells or primary microglia were subjected to escalating dosage of melatonin administration. The obtained LPS-treated BV-2 and primary microglia cells underwent the determination of iNOS, COX2, nuclear fraction (NF-κB (Ser536-p65) and Acetyl-NF-κB p65 (Lys310) and NF-κB activity. (A) Western blot measurement of iNOS and COX-2 in LPS treated BV-2 cells in relation to the escalating dosage of melatonin. (B) Western blot measurement of iNOS and COX-2 in LPS treated primary cultured microglia cells in relation to escalating dosage of melatonin. (C) Measurement of nuclear fraction of NF-κB (Ser536-p65) and Acetyl-NF-κB p65 (Lys310) in LPS treated BV-2 cells. (D) Measurement of nuclear fraction of NF-κB (Ser536-p65) and Acetyl-NF-κB p65 (Lys310) in LPS-treated primary microglia cells. (E) Illustration of immunohistochemistry staining showing nuclear translocation of a-NF-κB p65 in LPS-treated primary microglia cells. (F) Illustration of immunohistochemistry staining showing nuclear translocation of a-NF-κB p65 in LPS-treated BV-2 cells. (G) Determination of NF-κB binding activity in LPS-treated BV-2 cells measured by ELISA-based TransAM® NFκB kit. (H) Measurement of luciferase activity of LPS-treated BV-2 cells related to a-NF-κB p65. N=6 in the independent values. Data analysis was done by performing one-way analysis of variance test followed by post hoc Tukey's test. Statistical significance: *p < 0.05; **p < 0.01; ***p<0.001, compared to control group. Bar length= 20μm.

Melatonin's inhibition of iNOS and COX-2 expressions in microglia was assessed (Fig. 2A, B). To explore the regulation of iNOS and COX-2, we examined NF-κB subunit activation. LPS treatment significantly increased NF-κB (Ser536-p65) nuclear translocation, while melatonin reduced this translocation. Similarly, acetylation of RelA at Lys310 was decreased by melatonin (Fig. 2C-F). Melatonin also significantly inhibited NF-κB (p65) DNA-binding activity, as shown by an ELISA-based TransAM® NFκB kit (Fig. 2G) and luciferase reporter assays (Fig. 2H). These findings suggest that melatonin's anti-inflammatory function is closely linked to the suppression of NF-κB activity.

### Melatonin inhibited ROS production, induced antioxidant enzymes expressions, and activated Nrf2 signaling in BV2 microglia cells

**Figure 3:**
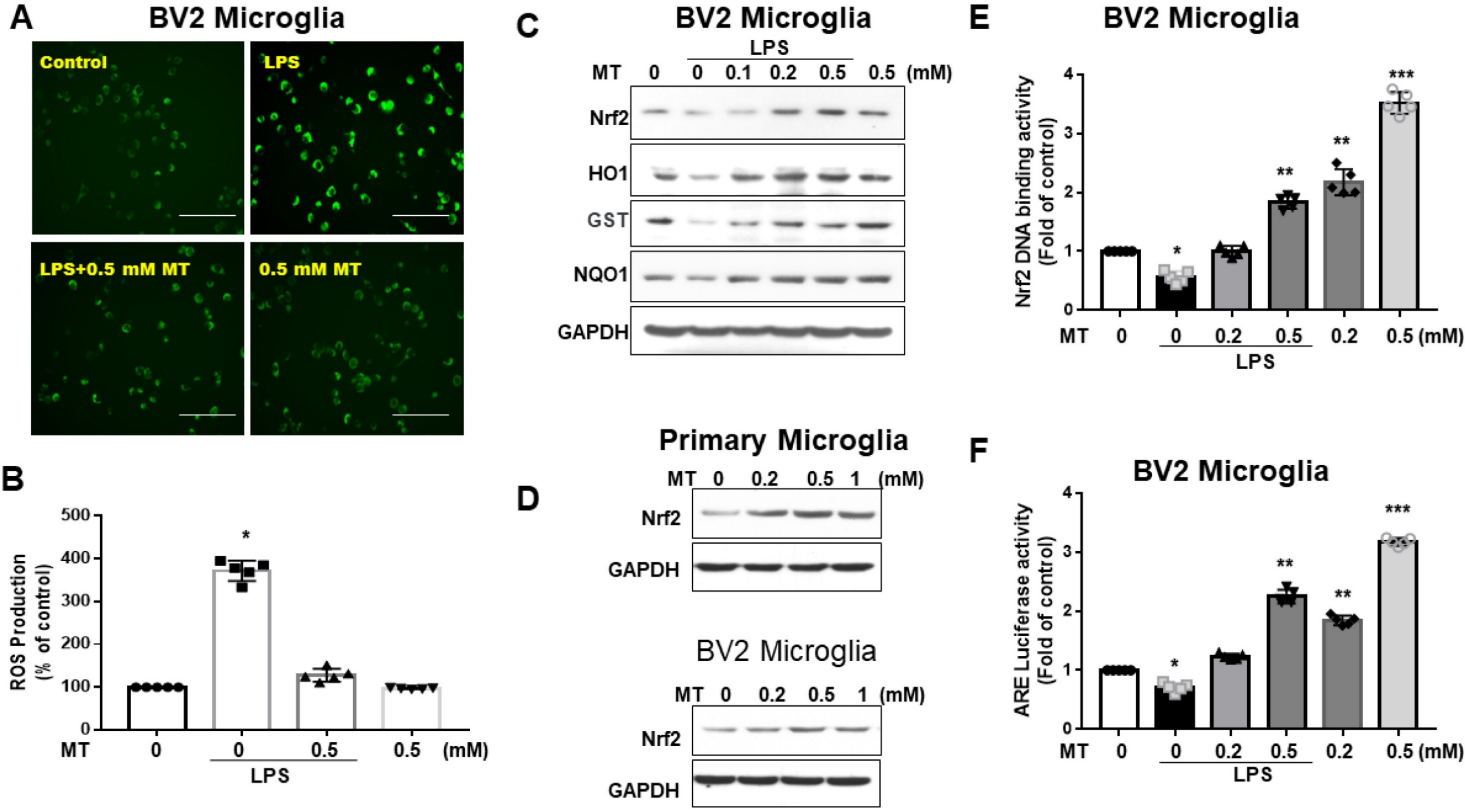
Attenuation of ROS and Nrf2 axis in LPS- treated microglia cells by melatonin administration. BV2 and primary microglia cells treated with LPS were subjected to the measurement of ROS, Nrf2, HO1, GST, NQO1. (A) Illustration of DCF fluorescence in LPS-treated BV-2 cells after melatonin administration. (B) Quantitative analysis in ROS production in LPS-treated BV-2 cells in relation to escalating dosage of melatonin. (C) Illustration of western blot results in LPS-treated BV-2 cells with escalating dosage of melatonin administration for Nrf2, HO1, GST, and NQO1, with GAPDH as the internal control. (D) Illustration of western blot analysis for Nrf2 in primary microglia cells and BV-2 cells, with GAPDH as the internal control. (E) Nrf2 DNA-binding activity in LPS-treated BV2 cells in relation to escalating dosage of melatonin. (F) Measurement of luciferase activity in the Nrf2 transcriptional activity by transfecting the cells with a plasmid containing ARE. N=6 independent values. Data analysis was done by performing one-way analysis of variance test followed by post hoc Tukey's test. Statistical significance: *p < 0.05; **p < 0.01; ***p<0.001, compared to control group. Bar length=100μm.

Melatonin significantly reduced DCF-positive cells in LPS-activated BV2 cells (Fig. 3A). Quantified DCF fluorescence assays showed that melatonin inhibited ROS production in a concentration-dependent manner (Fig. 3B). Western blot analysis revealed that melatonin activated the Nrf2-ARE signaling pathway, increasing protein expression of Nrf2, HO-1, GST, and NQO1 (Fig. 3C), and mRNA levels (data not shown). Melatonin also increased Nrf2 expression (Fig. 3D) and Nrf2 DNA-binding activity in BV2 cells (Fig. 3E). Luciferase reporter assays indicated that melatonin enhanced ARE-dependent luciferase activity dose-dependently (Fig. 3F). These findings suggest that melatonin's antioxidant activity is closely linked to increased nuclear accumulation and activation of Nrf2.

### *In silico* prediction in NF kappa-B domain linked to AhR using STRING, LIGPLOT and molecular docking

**Figure 4:**
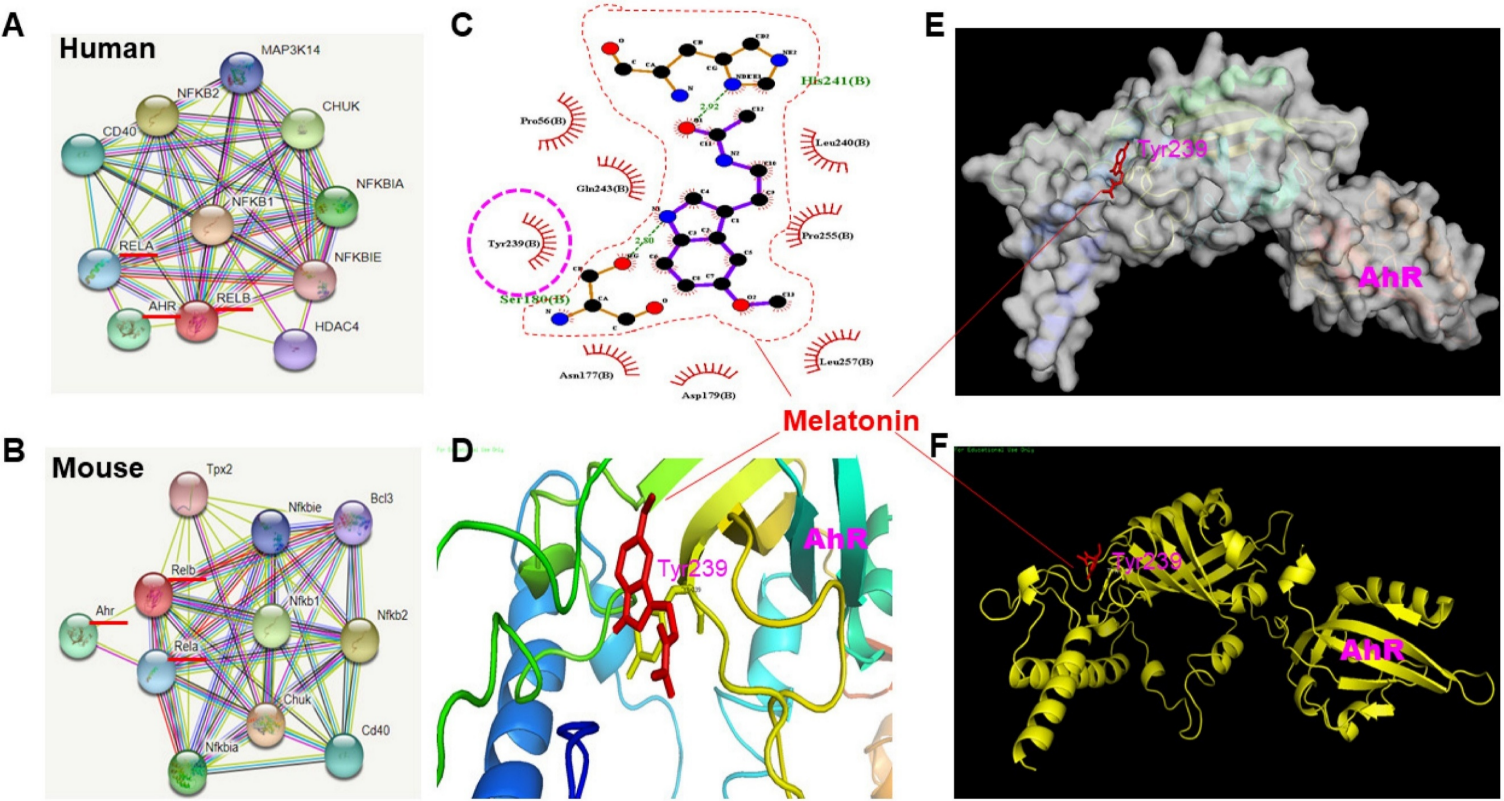
Prediction of the interaction between melatonin and AhR infrastructure using STRING, LIGPLOT and Molecular docking. To understand the infrastructure between melatonin and AhR, NFKB was used as the bait protein through STRING 9 to investigate the protein-protein interaction through functional enrichment analysis. Then, the protein-ligand interaction was depicted using LIGPLOT. Finally, the docking procedure was employed to predict the ligand conformation, position, and orientation to assess the binding capacity. (A) and (B) Schematic illustration showing the interaction of protein-protein predicted by STRING in human and mouse model, displaying the ten most confident interaction function based on the bait of NFKB. (C) The generation of schematic 2-D illustration of protein-ligand complex by LIGPLOT program from the standard protein data bank file. (D), (E), (F) Illustration of docking results in crystal structure (color ribbon), electrostatic potential surface diagram, and ribbon representation room, favoring AhR pocked site (Tyrosine 239) highly interacting with Melatonin.

Using STRING 9, the analysis showed a strong association between NFκB and AhR (Fig. 4A, B). Bioinformatics analysis via LIGPLOT identified Tyrosine 239 on AhR as a likely interaction site for melatonin (Fig. 4C). Docking studies, illustrated in Fig. 4D (color ribbons), Fig. 4E (electrostatic potential surface), and Fig. 4F (zoomed-in ribbon representation), revealed a high level of interaction between melatonin and the AhR pocket site at Tyrosine 239. To further validate the direct interaction between melatonin and AhR, we conducted a receptor-binding assay, competition assays, saturation curves, gene silencing techniques to knock down AhR expression, and investigate the effect of mutating Tyrosine 239 on AhR to verify that melatonin interacts specifically with this residue to test this hypothesis. The binding of [³H]-melatonin to HEK-293 cells expressing recombinant human AhR (Abbexa Ltd, Catalogue No: abx065464) during initial linearity studies using MES buffer is presented in Supplementary Figure 1. In this preparation, specific binding was nearly indistinguishable from total binding, with percent specific binding values of 97.7 ± 0.6% (n = 6) across the entire titration range. To optimize cell stock usage while maintaining an adequate signal-to-noise ratio, we determined that 11.6 mg per 0.1 ml of cell preparation would be sufficient for subsequent assays. Additionally, Krebs buffer was found to negatively impact [³H]-melatonin specific binding, leading to the selection of MES buffer for all further studies. We conducted competition binding assays using TCDD, a well-established AhR ligand, to validate the specificity of [^3^H]-Melatonin binding. As shown in Supplementary Figure 2, increasing concentrations of TCDD led to a dose-dependent reduction in [^3^H]-Melatonin binding to AhR, confirming competitive binding. The calculated IC₅₀ value for TCDD was approximately 1.37 nM, indicating strong affinity and specificity of the interaction. Saturation binding assays were performed and are presented in Supplementary Figure 3. Briefly, increasing concentrations of [^3^H]-Melatonin were incubated with a fixed amount of AhR, and specific binding was measured at each concentration. Non-specific binding was subtracted to yield specific binding values. From the resulting saturation curve, the calculated parameters were as follows: Bmax ≈ 104.37 (arbitrary units), indicating the maximum binding capacity. Kd ≈ 5.47 nM, representing the concentration at which 50% of AhR is occupied by [^3^H]-Melatonin. These results further support a high-affinity interaction between melatonin and AhR. We performed gene silencing experiments to reduce AhR expression and evaluate the impact on [^3^H]-Melatonin binding. As shown in Supplementary Figure 4, two different shRNA constructs (ShAhR #1 and ShAhR #2) effectively knocked down AhR expression. Consequently, [^3^H]-Melatonin binding was markedly reduced in AhR-deficient cells, supporting the specificity of the binding interaction. We introduced a point mutation converting Tyrosine 239 to Phenylalanine (Y239F) to assess the functional significance of this residue. This mutation removes the hydroxyl group while preserving the aromatic and hydrophobic nature of the side chain. As shown in Supplementary Figure 4, this mutation resulted in a notable reduction in [^3^H]-Melatonin binding to AhR, indicating that Tyr239 is likely critical for melatonin interaction, potentially via hydrogen bonding or phosphorylation-dependent mechanisms.

### Melatonin induced tyrosine 239 phosphorylation on AhR and reversed LPS-induced AhR degradation in activated cells of BV2 and primary microglia

**Figure 5:**
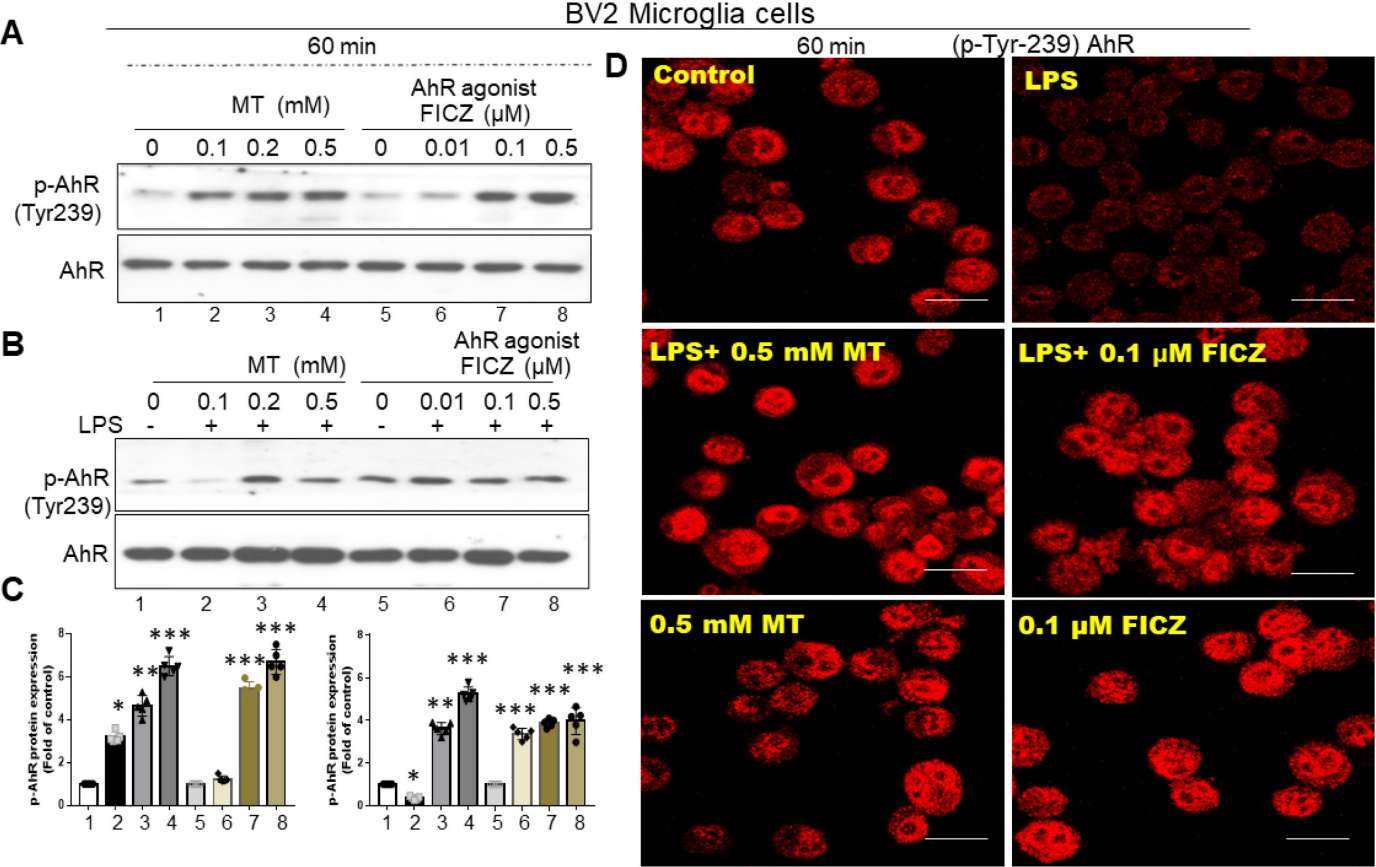
Restoration of AhR dephosphorylation in activated microglia by melatonin administration. Either primary microglia or BV-2 cells activated by LPS were subjected to the administration of melatonin or AhR agonist to assess the potential restoration of AhR dephosphorylation at the point of Tyr-239, determined by western blot and immunohistochemistry staining. (A) Illustration of western blot analysis of expression of AhR dephosphorylation (Tyr-239) in LPS-activated primary microglia cells administrated with escalating dosage of melatonin and AhR agonist (FICZ). (B) Illustration of western blot analysis of expression of AhR dephosphorylation (Tyr-239) in LPS-activated BV-2 cells administered by escalating dosage of melatonin and AhR agonist (FICZ). (C) Quantitative analysis of western blot results for p-AhR in (A) and (B). (D) Immunohistochemistry staining of p-tyrosine 239(AhR) in BV-cells treated with melatonin and FICZ. Data analysis was done by performing one-way analysis of variance test followed by post hoc Tukey's test. N=6. Statistical significance: *p < 0.05; **p < 0.01; ***p<0.001, compared to control group. Bar length= 20μm.

Based on in-silico predictions and docking procedures, the most likely melatonin binding site on AhR is Tyrosine 239. As shown in Fig. 5A and B, melatonin significantly induced phosphorylation at Tyrosine 239 on AhR at 60 minutes in both primary microglia cultures and BV-2 cells. Using 6-Formylindolo[3,2-b] carbazole (FICZ), an AhR ligand, as a positive control, we found that melatonin or FICZ completely inhibited LPS-induced AhR dephosphorylation at Tyr-239 (Fig. 5C). Immunohistochemistry revealed similar trends, showing LPS-induced AhR degradation in activated microglia over a longer time scale (Fig. 5D).

### AhR targeting Nrf2 promoter binding site

**Figure 6:**
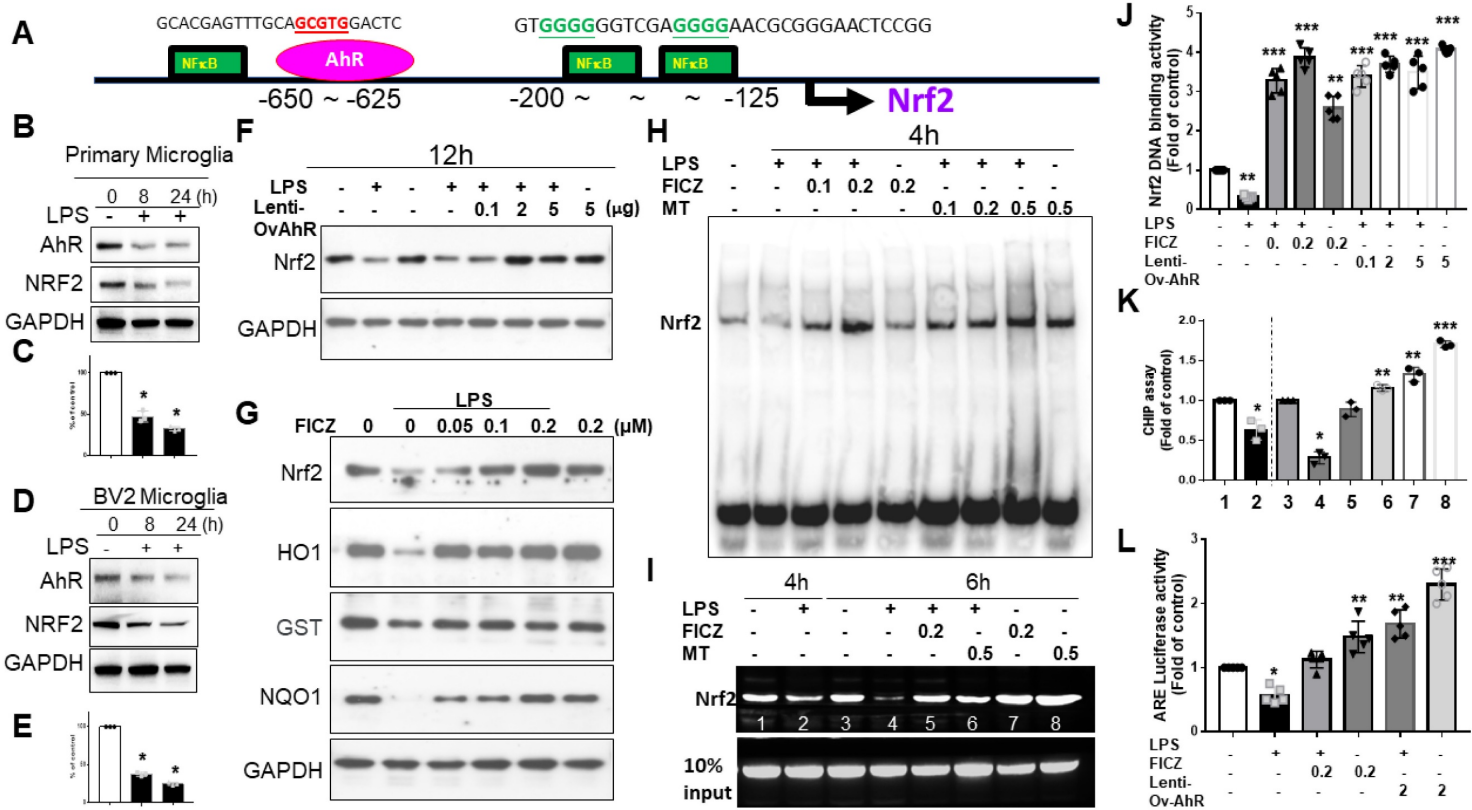
The AhR targeting on the Nrf2 promotor site in activated microglia cells. (A) In silico prediction of Nrf2 promoter binding site in AhR located between -650~-625 and NF-κB located between -200 -125. (B) Illustration of the co-expression of AhR and Nrf2 in activated primary microglia cells in relation to escalating dosage of LPS. (C) Quantitative analysis of western blot results in (B). (D) Illustration of the co-expression of AhR and Nrf2 in activated BV-2 cells in relation to escalating dosage of LPS. (E) Quantitative analysis of western blot results in (D). (F) Illustration of Nrf2 expression in LPS-activated BV-2 cells influenced by the lentivirus-carried AhR expression. (G) Illustration of Nrf2 and AhR-associated inflammatory gene (HO-1, GST, NQO-1) in LPS activated BV-2 cells after administration of escalating dosage of FICZ. (H) Illustration of EMSA in LPS-activated BV-2 cells treated with AhR agonist or melatonin at escalating dose. (I) The illustration of CHIP assay in LPS- activated BV-2 cells administered with escalating dosage of FICZ and melatonin at the time points of 4 and 6 hours. (J) Quantitative analysis of Nrf2 binding activity. (K) Quantitative analysis of CHIP assays. (L) Quantitative analysis of ARE luciferase activity. Data analysis was done by performing one-way analysis of variance test followed by post hoc Tukey's test. N=6. Statistical significance: *p < 0.05; **p < 0.01; ***p<0.001, compared to control group.

In silico prediction models showed AhR located at -650 to -625 and NFκB at -200 to -125 (Fig. 6A). LPS-activated primary microglia or BV2 cells displayed coupled down-regulation of AhR and Nrf2 (Fig. 6B-E). LPS-induced Nrf2 downregulation was reversed by lentivirus transfection of AhR (Fig. 6F). FICZ also counteracted Nrf2 downregulation, paralleling effects on HO-1, GST, and NQO-1 (Fig. 6G). In an EMSA, LPS decreased AhR-regulated Nrf2 binding, which was blocked by melatonin or FICZ at 4 hours. Melatonin or FICZ independently activated this binding activity (Fig. 6H). Lentivirus transfection of AhR or FICZ significantly increased Nrf2 DNA-binding activity in LPS-treated cells (Fig. 6J). ChIP assays showed that LPS-induced AhR-regulated Nrf2 degradation was reversed by melatonin or FICZ (Fig. 6I, K). Luciferase reporter assays revealed that transfection with AhR or FICZ significantly increased ARE-dependent luciferase activity, with FICZ exhibiting a similar pattern in ARE signaling cascade proteins (Fig. 6L). These findings suggest that Nrf2 regulation is closely linked to increased nuclear accumulation and activation of AhR.

### Lentivirus transfected AhR or FICZ thwarted pro-inflammatory mediator's production and promoted expression of anti-inflammatory M2 markers in LPS-stimulated BV2 microglia cells

**Figure 7:**
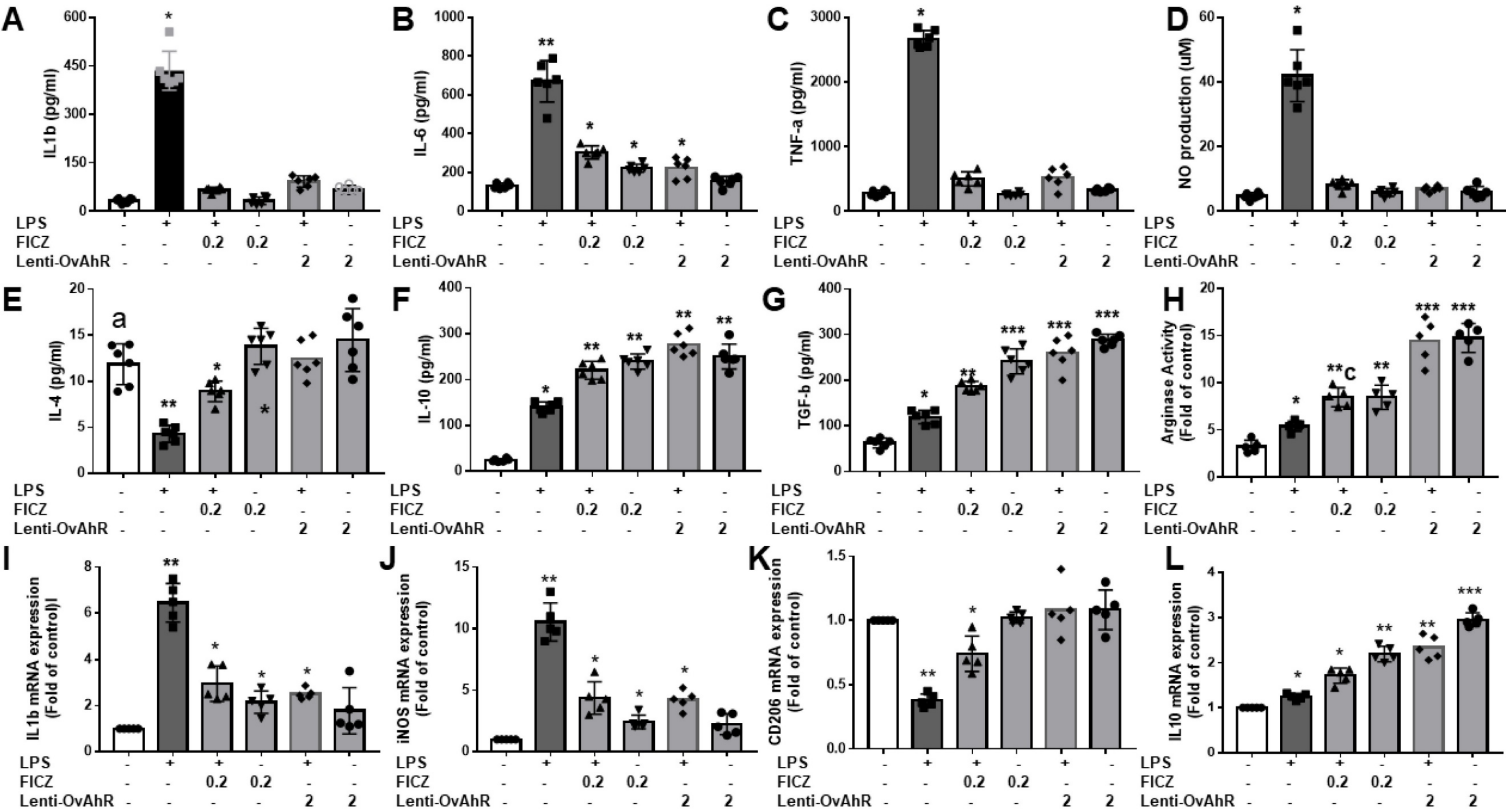
Attenuation of inflammatory response in LPS-activated microglia cells by the administration of AhR agonist or AhR transfection. The LPS-treated BV2 or primary microglia cells are subjected to an escalating dose of AhR agonist or lentivirus transfection of AhR. The supernatants were obtained for the determination of inflammatory cytokine (IL-1β, IL-6, TNF-α) and anti-inflammatory cytokines (IL-4, IL-10, TGF-β, arginase activity) by ELISA. The NO production was assessed by Griess assay. The harvested microglia cells were subjected to qRT-PCR to investigate the mRNA expression of IL-1β, iNOS, CD 206 and IL-10. (A) Determination of IL-1β expression in LPS treated BV2 in response to different dosage of FICZ or AhR transfection. (B) Determination of IL-6 expression in LPS treated BV2 in response to escalating dosage of FICZ or AhR transfection. (C) Determination of TNF-α expression in LPS treated BV2 in response to escalating dosage of FICZ or AhR transfection. (D) Measurement of NO production in LPS-treated BV2 cells in response to escalating dosage of FICZ or AhR transfection. (E) Determination of IL-4 expression in LPS- treated BV2 in response to escalating dosage of FICZ or AhR transfection. (F) Determination of IL-10 expression in LPS-treated BV2 cells in response to escalating dosage of FICZ or AhR transfection. (G) Determination of TGF-β expression in LPS-treated BV2 cells in response to escalating dosage of FICZ or AhR transfection. (H) Measurement of arginase activity in LPS- treated BV2 cells in response to escalating dosage of FICZ or AhR transfection. (I) Expression of Il-1β mRNA in LPS-treated BV2 cells in response to the escalating dosage of FICZ or AhR transfection, as a fold of increase related to control. (J) Expression of iNOS mRNA in LPS-treated BV2 cells in response to escalating dosage of FICZ or AhR transfection, measured as fold increase related to control. (K) Expression of CD 206 mRNA in LPS-treated primary cultured microglia cells in response to escalating dosage of FICZ or AhR transfection, measured as a fold increase related to control. (L) Expression of IL-10 mRNA in LPS-treated primary cultured microglia cells in response to escalating dosage of FICZ or AhR transfection, measured as a fold increase related to control. N=6 in each independent experiment. Data analysis was done by performing one-way analysis of variance test followed by post hoc Tukey's test. Statistical significance: *p < 0.05; **p < 0.01; ***p<0.001, compared to control group.

Based on ELISA and Griess assays, lentivirus-transfected AhR or FICZ inhibited LPS-induced production of pro-inflammatory cytokines (IL-1β, IL-6, and TNF-α) and reduced NO production in primary microglia and BV2 cells (Fig. 7A-D). Like melatonin, lentivirus transfection of AhR or FICZ also activated anti-inflammatory cytokines (IL-4, IL-10, and TGF-β) and increased arginase activity in LPS-stimulated BV2 cells (Fig. 7E-L). Additionally, lentivirus transfection of AhR or FICZ increased mRNA levels of M2 markers (CD206, IL-10) and decreased M1 markers (IL-1β, iNOS) in LPS-treated BV2 cells. These results suggest that targeting AhR activation reduces pro-inflammatory M1 mediators and increases anti-inflammatory M2 markers in LPS-stimulated microglia.

### Melatonin ameliorated neurological deficits in LPS microinjected animal model, paralleling the expression of AhR in activated microglia cells

**Figure 8:**
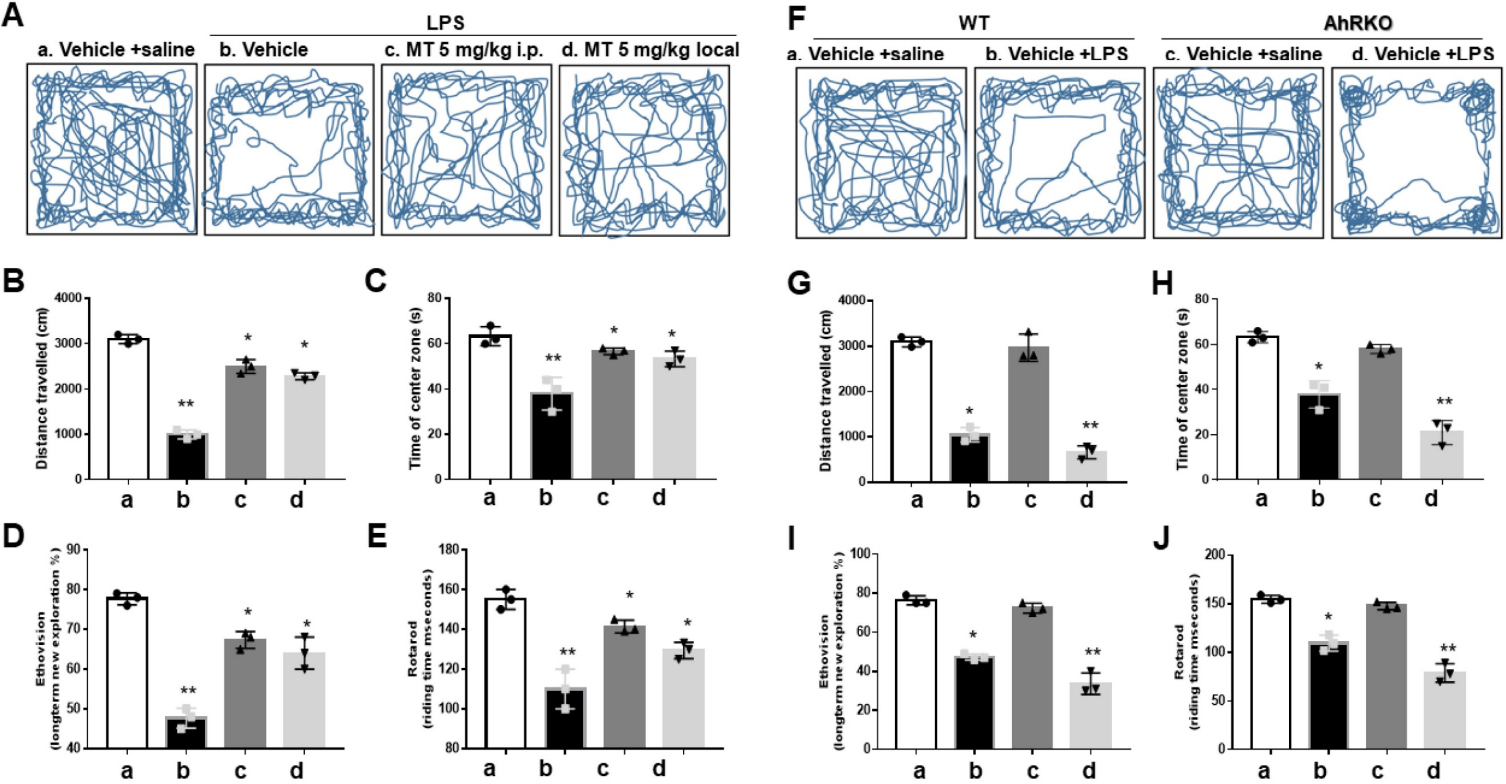
Improvement of neurobehavior in LPS-induced neuroinflammation by melatonin and worsened condition in AhR deletion. Either wild or AhR deleted mice were stereotactically microinjected with 5ug LPS or combined with melatonin 5mg with total 20 ul PBS at the coordinate of AP + 1.4 mm, ML -2.0 mm, DV -2.0 mm from bregma. After stereotactic microinjection, these animals underwent neurobehavioral assessment. (A) Illustration of the trajectory of locomotor activity in the different treatment groups. (B) Bar graph showing the total distance measured in cm for the different treatment groups. (C) Bar graph showing the time spent in the central area in the different treatment groups. (D) EthoVision plot of novel object exploration 24 hours after the new object change, presented as a percentage of new/old exploration. (E) Plot of retention time in Rotarod in the different treatment groups. (F) Illustration of the locomotor activity trajectory in AhR-deleted mice in the different treatment groups. (G) Bar graph showing the total distance measured in centimeters in AhR- deleted mice in the different treatment groups. (H) Bar graph showing the time spent in the central area in AhR-deleted mice in different treatment groups. (I) EthoVision plot of novel object exploration 24 hours in AhR deleted mice after the new object change, presented as percentage of new/old exploration. (J) Plot of retention time in Rotarod test in AhR-deleted mice related to the different treatment group. N=6. Data analysis was done by performing one-way analysis of variance test followed by post hoc Tukey's test. Statistical significance: *p < 0.05; **p < 0.01; ***p<0.001, compared to control group.

In the open-field test, melatonin pre-treatment significantly lessened the LPS-induced reduction in movement trajectories (Fig. 8A), total distance (Fig. 8B), and central time (Fig. 8C). The EthoVision assessment showed that melatonin alleviated the reduction in novel exploratory behaviors induced by LPS (Fig. 8D). Melatonin also improved the reduced retention time in the Rotarod test after LPS injection (Fig. 8E).

To investigate AhR function *in vivo*, AhR knockout (AhRKO) reduced LPS-induced locomotor activity, as evidenced by total movement trajectory (Fig. 8F), total distance (Fig. 8G), and central time (Fig. 8H). AhR deletion further aggravated the reduced retention time in the Rotarod test after LPS injections. Results from EthoVision (Fig. 8I) and Rotarod (Fig. 8J) were consistent.

**Figure 9:**
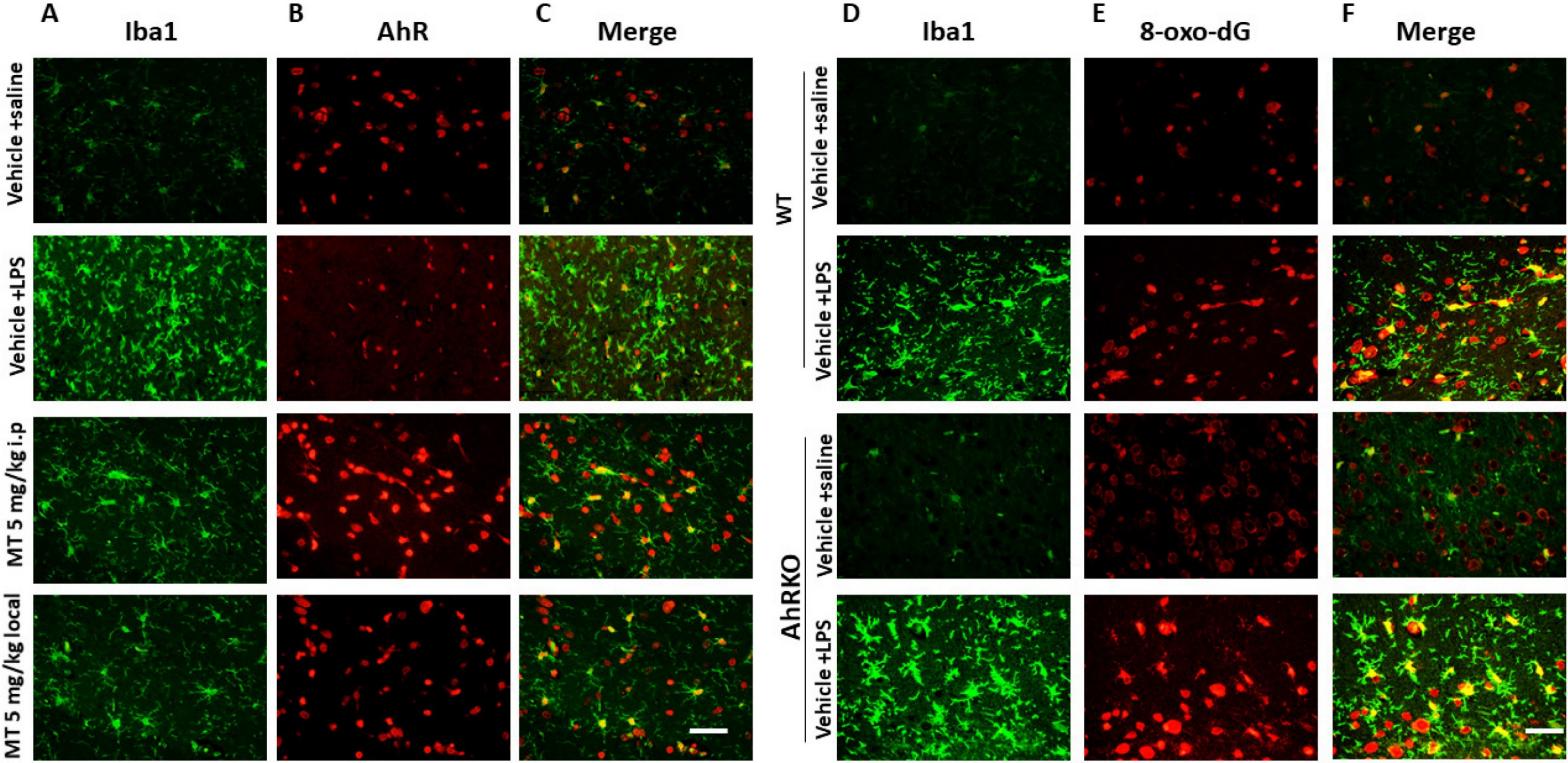
Immunohistochemistry staining of decreased microglial deposition activation with reciprocally increased AhR expression in LPS-induced neuroinflammation model administered by melatonin. Either wild or AhR- deleted mice were stereotactically microinjected with 5ug LPS or combined with melatonin 5mg with total 20 ul PBS at the coordinate of AP + 1.4 mm, ML -2.0 mm, DV -2.0 mm from bregma. These animals were sacrificed at 7 days after injury and allocated to different immunohistochemistry staining. (A) Illustration of activated microglia (Iba1) subjected to LPS injection in the hippocampus in the different treatment groups. (B) Illustration of AhR expression in the corresponding region. (C) Merged imaging of (A) and (B). (D) Illustration of activated microglia cells (positive Iba1) in the wild and AhR deleted mouse. (E) Expression of 8-ox-dG in the corresponding region. (F) Merged fusion of (D) and (E).

LPS injections increased the number of Iba1-positive cells, showing a de-ramified morphology with thicker processes and larger cell bodies, along with decreased AhR expressions in activated microglia. Melatonin significantly reduced the number of Iba1-positive cells, restored their morphology, and increased AhR expression (Fig. 9A-C). AhR deletion increased the number of activated microglia and triggered significant microglial death (Fig. 9D-F).

## Discussion

This study is the first to demonstrate melatonin's anti-inflammatory effects on activated microglia through phospho-AhR (Tyr239) activation, which upregulates Nrf2 via the AhR/Nrf2/ARE pathway. Using in silico methods like STRING, LIGPLOT, and molecular docking, we confirmed melatonin's interaction with phospho-AhR (Tyr239). Subsequent *in vitro* and *in vivo* experiments showed that melatonin upregulates Nrf2, modulating inflammation by suppressing pro-inflammatory and enhancing anti-inflammatory pathways. These findings suggest that combining melatonin with AhR agonists could effectively combat neuroinflammatory diseases.

The shift from M1 to M2 phenotype in activated microglia by melatonin is mainly due to a transition from M1 associated genes (iNOS, COX-2, CD86, IFNγg, IL-1βb, IL-6, TNF-α, CCL2) to M2 associated genes (IL-10, CD206, IL-4, Arginase-1, GM-CSF, IGF-1, TGF-β1, YM1/2,) 28. Melatonin lowers LPS-triggered production of pro-inflammatory cytokines like TNFα, IL-6, and IL1β 26, while mitigating mitochondrial toxicity, suppress apoptosis, and activating autophagy 27. Microglia can be triggered by LPS or IFN-γ to adopt an M1 phenotype, leading to expressing pro-inflammatory cytokines or through IL-4/IL-13 to adopt an M2 feature, facilitating inflammation decline, scavenging, and regenerative tissue repair 3, 37. Meanwhile, another activated M2 feature can generate the neuro-protective or neuro-supportive effects, forwarding recovery and enhancing brain repair or regeneration 38. In this study, the significant shift from of M1 to M2 phenotype was augmented by the melatonin administration in activated microglia, affecting gene transcription (mRNA), translation (Western blot), or function status (ELISA). Our present results are consistent with the literature described above.

In response to various stimuli, iNOS is considerably enhanced in various glial cells, such as astrocytes and microglia 39, 40. Its expression is induced or tigered by proinflammatory cytokines and/or bacterial lipopolysaccharide (LPS) 41, 42. NO synthesis catalyzed by iNOS in activated macrophages is critical for non-specific immunity 43, 44. Activated microglia produce inflammatory mediators like TNF-α and neurotoxic factors such as NO and prostaglandins via iNOS and COX-2 45-47. NF-κB is a key transcription factor that responds to harmful stimuli by inducing genes involved in immunity 48, inflammation, and stress, including NO, TNF-α, iNOS, COX-2, PLA2 and heat shock proteins (HSPs) 49-53. Activated by ROS/RNS, NF-κB promotes the pro-inflammatory genes like COX-2, TNF-α, iNOS, MMP 9, HO-1, interleukin (IL)-1β and IL-6, leading to tissue injury and apoptosis 53, 54. These cytokines further activate NF-κB, creating a positive feedback loop that amplifies inflammation 51, 54. In this study, the LPS-activated microglia showed robust responses in terms of iNOS and COX2 expressions, which were attenuated by melatonin treatment. The modulation of such inflammatory response was also consistent with the modulation of NF-κB expression, whether in terms of binding capacity, nuclear translocation, transcription, or translation.

Nuclear translocation of NF-κBp65 is a response to ROS and cytokine stimulations, driven by NF-κB mediated transcriptional activation 55. This process is promoted by phosphorylation of Ser-536 in p65 by kinases such as IKK, RSK1, and TBK1, and further facilitated by CDK-6 in the nucleus, which aids in binding to specific promoter sequences 56-58. Inhibiting CDK-6 suppresses NF-κB p65-mediated inflammatory gene expressions 59, 60. Acetylation, particularly at Lys310, is crucial for full transcriptional activity of NF-κB p65 61, 62. We measured the entire levels of NF-κB p65 protein, as well as the phosphorylation (Ser 536) and acetylation (Lys 310) of NF-κB p65 in the nuclear and cytosolic fractions of LPS-activated primary microglia or BV-2 cells. Melatonin treatment resulted in a significant drop in NF-κBp65 levels in the nucleus relative to the cytosol, suggesting a reduction of NF-κB pathway activation after melatonin treatment. Using ELISA to measure binding capacity and luciferase activity, we observed a significant decrease in NF-κB p65 mRNA expression following melatonin treatment compared to controls. These findings suggest that LPS-induced microglial activation involves NF-κB activation, nuclear translocation, and increased NF-κB gene expression. Melatonin downregulates all these processes.

Nrf2, a versatile transcription factor found abundantly in microglia, activates antioxidants such as heme oxygenase (HO-1), superoxide dismutase, catalase, glutathione sulfhydryl transferase, and haptoglobin (Hp) 63. It plays a crucial role in regulating microglial function in stroke and neurodegenerative diseases 64-66. Upregulating Nrf2 suppresses NF-κB and facilitates the transformation of microglial into an anti-inflammatory phenotype 67, 68, thereby activating the transcription of various genes containing antioxidant response element (ARE) in their promoter regions 69. In this study, LPS activated microglia to exert the oxidative stress as shown by increased DCF fluorescence and ROS production, all of which were attenuated by melatonin. These effects are primarily due to increased anti-oxidant reactions, including Nrf-2, HO-1, GCS and NQO-1 as well as enhanced Nrf2 DNA binding ability and ARE luciferase activity. These findings implied that melatonin had modulated the oxidative stress in LPS activated microglia through key factors involving Nrf-2.

The Nrf2 and NF-κB pathways mutually regulate responses to oxidative stress and inflammation. NF-κB's p65 subunit directly inhibits Nrf2 at the transcriptional level by competing for the CH1-KIX domain of the co-activator CBP, thus deactivating Nrf2 signaling 70. NF-κB also recruits HDAC3, leading to local hypoacetylation and further impairing Nrf2 activity 71, 72. Interestingly, certain anti-inflammatory agents activate Nrf2 by modulating NF-κB activity 73-76. In Nrf2-deficient mice with severe head injury, NF-κB activity is significantly higher compared to wild-type mice 77. Studies on primary cultured astrocytes from Nrf2 wild-type and knockout mice show elevated NF-κB activity and inflammatory cytokine expression in Nrf2 knockout cells, particularly TNF-α, IL-1β, IL-6, and MMP9 78. Conversely, NF-κB-DNA-binding activity is reduced in a diabetic mouse model overexpressing Nrf2 79. Our findings on the reciprocal regulation of NF-κB and Nrf2 in LPS-activated microglia treated with melatonin align with existing literature.

The aryl hydrocarbon receptor (AhR) is a potential receptor for melatonin due to their structural similarities, involving tryptophan metabolites and indolic compounds 80-82. AhR can be activated by a range of synthetic and natural chemicals, including halogenated aromatic hydrocarbons (HAHs) and nonhalogenated polycyclic aromatic hydrocarbons (PAHs) 83, 84. AhR's ligand-binding domain (LBD) binds diverse chemicals such as bilirubin, arachidonic acid, lipoxin A4 metabolites, prostaglandin G derivatives, and tryptophan derivatives like indigo dye, indole acetic acid, and indirubin 80-82, 85, 86. Melatonin and its metabolites can act as agonists on AhR in keratinocytes 87. Our study found that increasing melatonin doses enhanced phosphorylation of AhR at tyrosine 239 in LPS-activated microglia cells. Melatonin's effects were similar to AhR agonists or AhR transfection in modulating anti-inflammatory responses and Nrf2 expression.

AhR plays a key role in activating Nrf2, a transcription factor crucial for defending against oxidative stress. AhR likely binds to a xenobiotic response element (XRE) in the NRF2 gene locus to enhance NRF2 expression and facilitate its antioxidant effects 88, 89. Pharmacological activation of Nrf2 induces AhR mRNA and the expression of target genes like Cyp1a1 and Cyp1b1, thereby showing direct regulation of AhR transcription by Nrf2. Luciferase and chromatin immunoprecipitation (ChIP) assays confirm that Nrf2 binds to a CsMBE at -230 in the AhR promoter 21. The AhR ligand TCDD induces Nrf2 mRNA expression in an AhR-dependent manner, suggesting AhR directly enhances Nrf2 transcription 90. Additionally, Nrf2 activation by oxidative stress may occur through induction of CYPs via NQO1 gene expression 91. In an AhR-dependent manner, Nrf2 activity induces target genes such as Nqo1 and Gsta1 89. This study demonstrates that AhR agonists or AhR transfection significantly increase AhR expression in activated microglia, supporting the hypothesis that AhR can mitigate oxidative stress by enhancing Nrf2 expression.

The CA1 and CA3 sectors of the hippocampus proper serve distinct roles in spatial and contextual memories 92, 93. Pharmacological inactivation or neurotoxic lesions in animal models are commonly used to study contextual memory encoding and retrieval 6, 7, 32, 94, 95. The CA3 sector is crucial for spatial pattern association, completion, novelty detection, and short-term memory. Meanwhile, the CA1 sector acts as a "novelty" detector, identifying discrepancies between inputs from the entorhinal cortex and CA3 sector 6, 7, 95. In novel object tests using EthoVision XT, both recent and remote memories are impaired by hippocampal damage but can be improved with pharmacological interventions 6, 95. In this study, AhR deficiency was detrimental to neurological functions, affecting locomotor and memory function. Melatonin mitigated these deficits, correlating with increased AhR expression and reduced microglial oxidative stress.

There were several limitations in this study. The findings primarily stem from *in vitro* experiments, and their relevance needs validation in *in vivo* models and diverse neuroinflammatory conditions. While the study attributes melatonin's effects to AhR activation and the Nrf2/ARE pathway, it does not explore other potential off-target effects of melatonin or the role of alternative pathways. The focus on phospho-AhR (Tyr239) may overlook other regulatory mechanisms of AhR, and the variability in microglial activation states across different diseases might limit generalizability. Furthermore, translational challenges, such as optimal dosing, timing, and side effects, remain unaddressed. A comparative analysis with existing therapies and exploration of other pathways influencing microglia would strengthen the therapeutic implications of these findings.

## Conclusion

Melatonin suppressed NF-κB activity, reduced pro-inflammatory mediator production, and promoted an M2 phenotype in activated microglia. It also demonstrated potent antioxidant effects through activation of phospho-AhR (Tyr239) and subsequent upregulation of Nrf2 via the AhR/Nrf2/ARE pathway. These findings suggest that melatonin, in collaboration with AhR, holds therapeutic promise for neuroinflammatory disorders.

## Supplementary Material

Supplementary figures.

## Abbreviations

AhR: Aryl hydrocarbon receptor
AhRKO: AhR knockout
Nrf2: Erythroid 2-related factor 2
ARE: antioxidant response element
LPS: Lipopolysaccharide
ROS: Reactive oxygen species
NF-κB: Nuclear factor kappa-light-chain-enhancer of activated B cells
PAS: Per-Arnt-Sim
HSP90: heat shock protein 90
XAP2: X-associated cellular protein 2
pp60src: Rous sarcoma virus (RSV)-transforming protein
TCDD: 2,3,7,8-Tetrachlorodibenzo-P-dioxin
HO-1: heme oxygenase-1
NQO1: NAD(P)H-Quinone Oxidoreductase 1
GST: Glutathione S-transferase
SIRT1: Sirtuin 1
EMSA: Electrophoretic mobility shift assay
