# Supplementary material for: Melatonin Ameliorate neuroinflammation in activated microglia through the Aryl hydrocarbon-Nrf2 axis
Source: Int J Biol Sci. 2025 Jun 9;21(9):3917–33. doi: 10.7150/ijbs.105081 (PMC12210381; doi:10.7150/ijbs.105081)
Supplement: Supplementary file 1 — Supplementary figures. [file ijbsv21p3917s1.pdf]

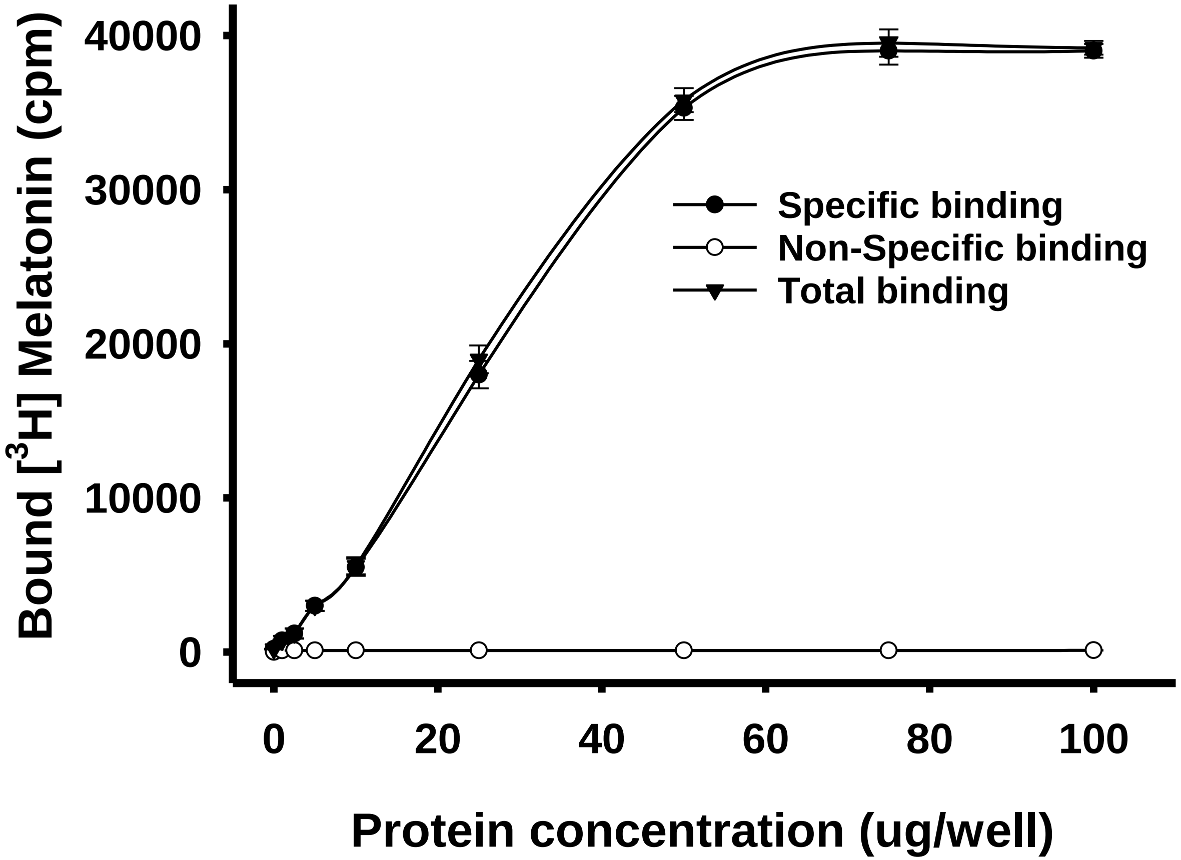

Pharmacological characterization of [^{3}H]-Melatonin binding to AhR. The binding of [^{3}H]-Melatonin to HEK-293 cell containing the recombinant human AhR (Abbexa Ltd, Catalogue No: abx065464) during initial linearity studies using MES buffer is shown in Supplementary Figure 1. Specific binding was nearly indistinguishable from total binding in this preparation, with percent specific binding values of 97.7 ± 0.6% (n = 6) across the entire titration range. To conserve cell stocks while maintaining an adequate signal-to-noise ratio, it was determined that 11.6 mg per 0.1 ml of cell preparation would be sufficient for subsequent assays. Krebs buffer was found to be detrimental to specific [^{3}H]-Melatonin binding, leading to the selection of MES buffer for all further studies.

Supplementary Fig. 1 Pharmacological characterization of [^{3}H]-Melatonin binding to AhR. Biological tissue linearity of [^{3}H]-Melatonin binding to HEK-293 cell membranes expressing the recombinant human AhR. Samples were added in varying amounts to [^{3}H]-Melatonin in a final volume of 500 µl and the binding assay was performed as described in the "Materials and methods" section. Data from a representative experiment are shown. Note the very high level of specific binding found in this system. All the results were expressed at least three independent experiments and presented.

Competition Assays:

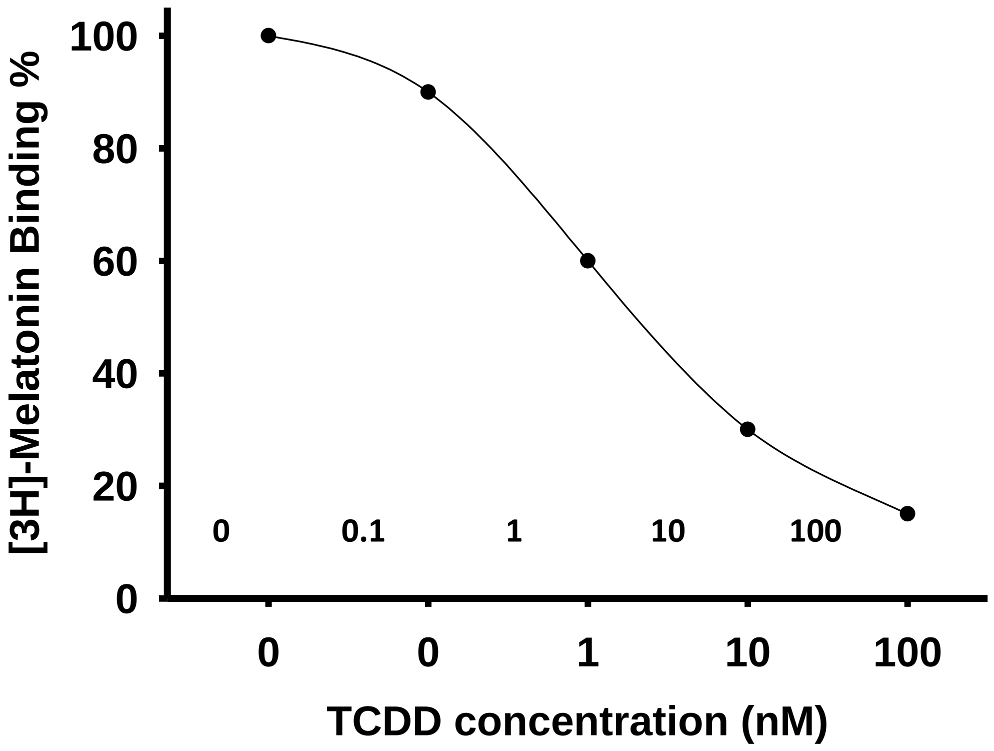

Result description:

Competitive binding assays were performed using [^{3}H]-melatonin as a tracer, as described in the Materials and Methods. TCDD was added to the reactions at the final concentrations indicated on the x-axis. Each data point represents the mean of three replicates. The data shown are representative of three independent experiments. The calculated IC_{50} for TCDD in this assay is approximately 1.37 nM, indicating the concentration at which [^{3}H]-melatonin binding to AhR is reduced by 50%, and thus demonstrating strong competitive binding. The competition assay plot showing markedly increasing concentrations of TCDD reduce [^{3}H]-Melatonin binding to AhR, indicating competitive binding and supporting specificity.

Supplementary Fig. 2 Competition assays confirming the specificity of [^{3}H]-Melatonin binding to AhR using known AhR ligands by TCDD. Competitive binding assays were performed using [^{3}H]-melatonin as a tracer, as described in the Materials and Methods. TCDD was added to the reactions at the final concentrations indicated on the x-axis. Each data point represents the mean of three replicates. The data shown are representative of three independent experiments. The calculated IC_{50} for TCDD in this assay is approximately 1.37 nM, indicating the concentration at which [^{3}H]-melatonin binding to AhR is reduced by 50%, and thus demonstrating strong competitive binding.

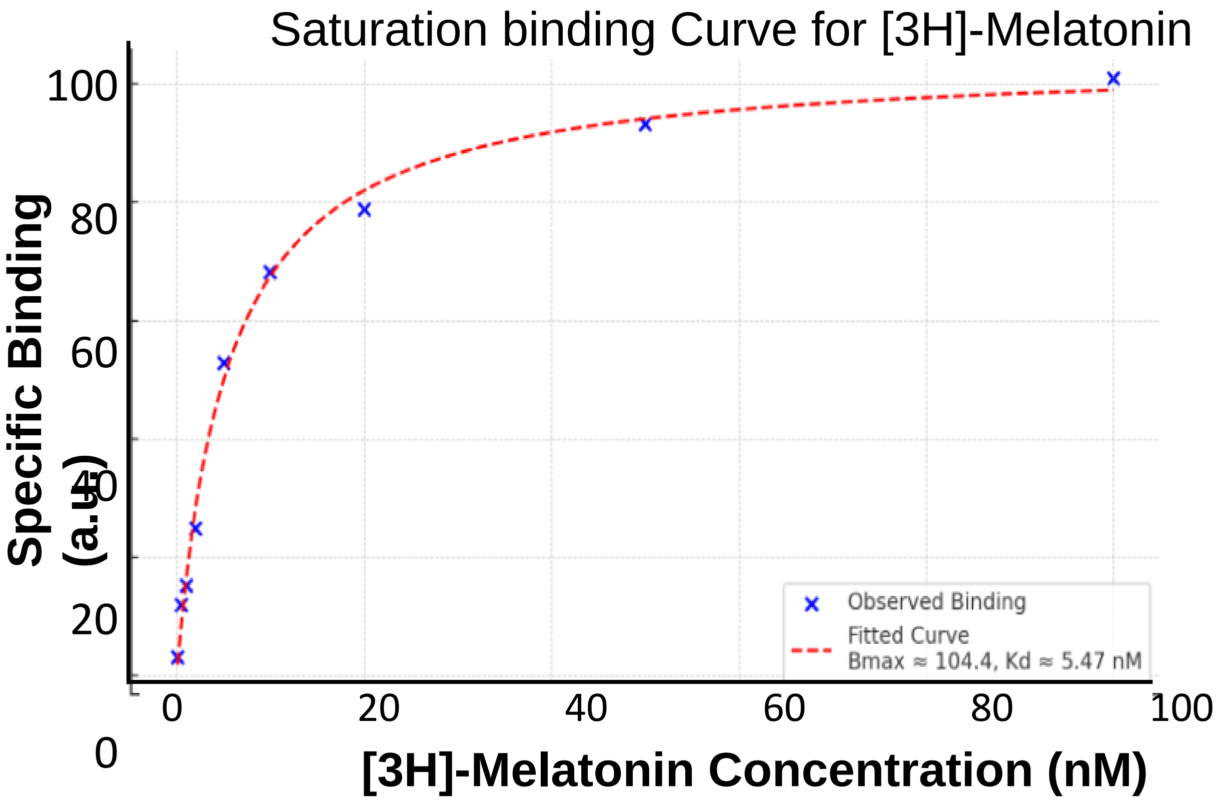

Result description:

[^3H]-Melatonin Exhibits Specific and High-Affinity Binding to AhR

To determine the dissociation constant (Kd) and maximum binding capacity (Bmax) for [^{3}H]-Melatonin, we perform a saturation binding assay and fit the data to the Langmuir binding isotherm (one-site specific binding model). Saturation binding assays revealed that [^{3}H]-melatonin binds specifically to the AhR with moderate to high affinity. Nonlinear regression analysis of the binding curve yielded a dissociation constant (Kd) of approximately 5.47 nM and a maximum binding capacity (Bmax) of ~104.37 arbitrary units. These values confirm a specific and saturable interaction between melatonin and AhR.

Supplementary Fig. 3. Saturation binding curve for [^{3}H]-melatonin binding to AhR. Saturation binding assays were performed to determine the maximum binding capacity (Bmax) and the dissociation constant (Kd) of [^3H]-melatonin for the aryl hydrocarbon receptor (AhR). Increasing concentrations of [^{3}H]-melatonin were incubated with AhR-containing cytosolic extracts under equilibrium binding conditions. Specific binding was determined by subtracting nonspecific binding (measured in the presence of excess unlabeled melatonin) from total binding. Nonlinear regression analysis yielded a Bmax of approximately 104.37 (arbitrary units) and a Kd of approximately 5.47 nM, indicating moderate to high affinity of [^{3}H]-melatonin for AhR.

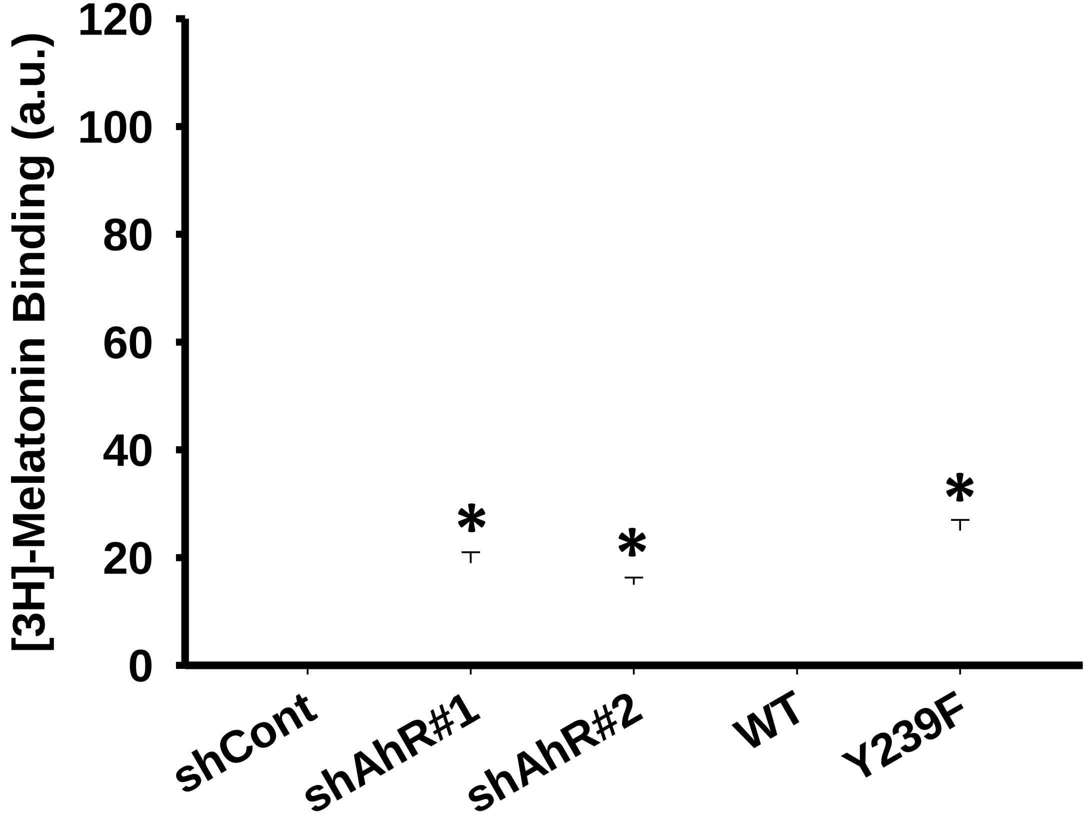

Result description:

Validation of [^3H]-Melatonin Binding Specificity via AhR Knockdown and Y239 Mutation

To confirm the specificity of [^{3}H]-melatonin binding to the AhR, we performed binding assays using knockdown AhR silenced cells. In shAhR#1, [^{3}H]-melatonin binding was nearly abolished, demonstrating that binding is strictly dependent on the presence of AhR. Similarly, knockdown of AhR expression using shAhR#2 cells resulted in a substantial reduction in binding, further supporting AhR's role in mediating the interaction.

To evaluate the role of Tyr239 in melatonin binding, we generated a Y239F mutant of AhR through gene synthesis mutagenesis. Although the tyrosine (Y)-to-phenylalanine (F) substitution is considered a conservative mutation, [^{3}H]-melatonin binding was significantly reduced in cells expressing the Y239F mutant compared to wild-type AhR. This reduction indicates that Tyr239 is critical for the interaction between melatonin and AhR, likely contributing to direct contact or conformational integrity at the binding interface. These findings collectively validate that [^{3}H]-melatonin binds specifically and directly to AhR, with Tyr239 playing a key role in ligand recognition.

Supplementary Fig. 4. Validation of [^{3}H]-melatonin binding specificity using AhR-knockdown cells and Tyr239 mutant AhR. To confirm the specific binding of [^{3}H]-melatonin to the aryl hydrocarbon receptor (AhR), binding assays were performed in shRNA controls (shNC) include the pLKO.1 empty vector and shAhR#1 and shAhR#2 cells. A marked reduction in [^{3}H]-melatonin binding was observed in both shAhR#1 and shAhR#2 cells, indicating the dependence of binding on AhR expression. Additionally, gene synthesis was used to replace tyrosine 239 with phenylalanine (Y239F) in AhR. Cells expressing the Y239F mutant exhibited significantly reduced [^{3}H]-melatonin binding compared to wild-type AhR, suggesting that Tyr239 plays a critical role in melatonin–AhR interaction. Data are representative of at least three independent experiments.
